# LUSTR: a new customizable tool for calling genome-wide germline and somatic short tandem repeat variants

**DOI:** 10.1186/s12864-023-09935-9

**Published:** 2024-01-26

**Authors:** Jinfeng Lu, Camilo Toro, David R. Adams, Undiagnosed Diseases Network, Maria T. Acosta, Margaret Adam, Raquel L. Alvarez, Justin Alvey, Laura Amendola, Ashley Andrews, Euan A. Ashley, Carlos A. Bacino, Guney Bademci, Ashok Balasubramanyam, Dustin Baldridge, Jim Bale, Michael Bamshad, Deborah Barbouth, Pinar Bayrak-Toydemir, Anita Beck, Alan H. Beggs, Edward Behrens, Gill Bejerano, Hugo J. Bellen, Jimmy Bennett, Beverly Berg-Rood, Jonathan A. Bernstein, Gerard T. Berry, Anna Bican, Stephanie Bivona, Elizabeth Blue, John Bohnsack, Devon Bonner, Lorenzo Botto, Brenna Boyd, Lauren C. Briere, Gabrielle Brown, Elizabeth A. Burke, Lindsay C. Burrage, Manish J. Butte, Peter Byers, William E. Byrd, John Carey, Olveen Carrasquillo, Thomas Cassini, Ta Chen Peter Chang, Sirisak Chanprasert, Hsiao-Tuan Chao, Ivan Chinn, Gary D. Clark, Terra R. Coakley, Laurel A. Cobban, Joy D. Cogan, Matthew Coggins, F. Sessions Cole, Heather A. Colley, Heidi Cope, Rosario Corona, William J. Craigen, Andrew B. Crouse, Michael Cunningham, Precilla D’Souza, Hongzheng Dai, Surendra Dasari, Joie Davis, Jyoti G. Dayal, Esteban C. Dell’Angelica, Patricia Dickson, Katrina Dipple, Daniel Doherty, Naghmeh Dorrani, Argenia L. Doss, Emilie D. Douine, Dawn Earl, David J. Eckstein, Lisa T. Emrick, Christine M. Eng, Marni Falk, Elizabeth L. Fieg, Paul G. Fisher, Brent L. Fogel, Irman Forghani, William A. Gahl, Ian Glass, Bernadette Gochuico, Page C. Goddard, Rena A. Godfrey, Katie Golden-Grant, Alana Grajewski, Don Hadley, Sihoun Hahn, Meghan C. Halley, Rizwan Hamid, Kelly Hassey, Nichole Hayes, Frances High, Anne Hing, Fuki M. Hisama, Ingrid A. Holm, Jason Hom, Martha Horike-Pyne, Alden Huang, Sarah Hutchison, Wendy Introne, Rosario Isasi, Kosuke Izumi, Fariha Jamal, Gail P. Jarvik, Jeffrey Jarvik, Suman Jayadev, Orpa Jean-Marie, Vaidehi Jobanputra, Lefkothea Karaviti, Shamika Ketkar, Dana Kiley, Gonench Kilich, Shilpa N. Kobren, Isaac S. Kohane, Jennefer N. Kohler, Susan Korrick, Mary Kozuira, Deborah Krakow, Donna M. Krasnewich, Elijah Kravets, Seema R. Lalani, Byron Lam, Christina Lam, Brendan C. Lanpher, Ian R. Lanza, Kimberly LeBlanc, Brendan H. Lee, Roy Levitt, Richard A. Lewis, Pengfei Liu, Xue Zhong Liu, Nicola Longo, Sandra K. Loo, Joseph Loscalzo, Richard L. Maas, Ellen F. Macnamara, Calum A. MacRae, Valerie V. Maduro, AudreyStephannie Maghiro, Rachel Mahoney, May Christine V. Malicdan, Laura A. Mamounas, Teri A. Manolio, Rong Mao, Kenneth Maravilla, Ronit Marom, Gabor Marth, Beth A. Martin, Martin G. Martin, Julian A. Martínez-Agosto, Shruti Marwaha, Jacob McCauley, Allyn McConkie-Rosell, Alexa T. McCray, Elisabeth McGee, Heather Mefford, J. Lawrence Merritt, Matthew Might, Ghayda Mirzaa, Eva Morava, Paolo Moretti, John Mulvihill, Mariko Nakano-Okuno, Stanley F. Nelson, John H. Newman, Sarah K. Nicholas, Deborah Nickerson, Shirley Nieves-Rodriguez, Donna Novacic, Devin Oglesbee, James P. Orengo, Laura Pace, Stephen Pak, J. Carl Pallais, Christina G. S. Palmer, Jeanette C. Papp, Neil H. Parker, John A. Phillips, Jennifer E. Posey, Lorraine Potocki, Barbara N. Pusey Swerdzewski, Aaron Quinlan, Deepak A. Rao, Anna Raper, Wendy Raskind, Genecee Renteria, Chloe M. Reuter, Lynette Rives, Amy K. Robertson, Lance H. Rodan, Jill A. Rosenfeld, Natalie Rosenwasser, Francis Rossignol, Maura Ruzhnikov, Ralph Sacco, Jacinda B. Sampson, Mario Saporta, Judy Schaechter, Timothy Schedl, Kelly Schoch, Daryl A. Scott, C. Ron Scott, Elaine Seto, Vandana Shashi, Jimann Shin, Edwin K. Silverman, Janet S. Sinsheimer, Kathy Sisco, Edward C. Smith, Kevin S. Smith, Lilianna Solnica-Krezel, Ben Solomon, Rebecca C. Spillmann, Joan M. Stoler, Kathleen Sullivan, Jennifer A. Sullivan, Angela Sun, Shirley Sutton, David A. Sweetser, Virginia Sybert, Holly K. Tabor, Queenie K.-G. Tan, Amelia L. M. Tan, Arjun Tarakad, Mustafa Tekin, Fred Telischi, Willa Thorson, Cynthia J. Tifft, Alyssa A. Tran, Rachel A. Ungar, Tiina K. Urv, Adeline Vanderver, Matt Velinder, Dave Viskochil, Tiphanie P. Vogel, Colleen E. Wahl, Melissa Walker, Stephanie Wallace, Nicole M. Walley, Jennifer Wambach, Jijun Wan, Lee-kai Wang, Michael F. Wangler, Patricia A. Ward, Daniel Wegner, Monika Weisz Hubshman, Mark Wener, Tara Wenger, Monte Westerfield, Matthew T. Wheeler, Jordan Whitlock, Lynne A. Wolfe, Kim Worley, Changrui Xiao, Shinya Yamamoto, John Yang, Zhe Zhang, Stephan Zuchner, Cristiane Araujo Martins Moreno, Wan-Ping Lee, Yuk Yee Leung, Mathew B. Harms, Badri Vardarajan, Erin L. Heinzen

**Affiliations:** 1 Division of Pharmacotherapy and Experimental Therapeutics, Eshelman School of Pharmacy, University of North Carolina at Chapel Hill, Chapel Hill, NC 27599 USA; 2 NIH Undiagnosed Diseases Program, National Human Genome Research Institute (NHGRI), National Institutes of Health, Bethesda, MD 20892 USA; 3 Neurology Department, Universidade de São Paulo, São Paulo, SP 05508-010 Brazil; 4 Penn Neurodegeneration Genomics Center, Department of Pathology and Laboratory MedicinePerelman School of Medicine, University of Pennsylvania, Philadelphia, PA 19104 USA; 5 Department of Neurology, Division of Neuromuscular Medicine, Columbia University Irving Medical Center, New York, NY 10032 USA; 6 The Taub Institute for Research On Alzheimer’s Disease and the Aging Brain, Gertrude H. Sergievsky Center, Department of Neurology, College of Physicians and Surgeons, Columbia University, The New York Presbyterian Hospital, New York, NY 10032 USA; 7 Department of Genetics, School of Medicine, University of North Carolina at Chapel Hill, Chapel Hill, NC 27599 USA

**Keywords:** Short tandem repeats, Bioinformatics, Variant calling tool kit, Somatic, LUSTR

## Abstract

**Background:**

Short tandem repeats (STRs) are widely distributed across the human genome and are associated with numerous neurological disorders. However, the extent that STRs contribute to disease is likely under-estimated because of the challenges calling these variants in short read next generation sequencing data. Several computational tools have been developed for STR variant calling, but none fully address all of the complexities associated with this variant class.

**Results:**

Here we introduce LUSTR which is designed to address some of the challenges associated with STR variant calling by enabling more flexibility in defining STR loci, allowing for customizable modules to tailor analyses, and expanding the capability to call somatic and multiallelic STR variants. LUSTR is a user-friendly and easily customizable tool for targeted or unbiased genome-wide STR variant screening that can use either predefined or novel genome builds. Using both simulated and real data sets, we demonstrated that LUSTR accurately infers germline and somatic STR expansions in individuals with and without diseases.

**Conclusions:**

LUSTR offers a powerful and user-friendly approach that allows for the identification of STR variants and can facilitate more comprehensive studies evaluating the role of pathogenic STR variants across human diseases.

**Supplementary Information:**

The online version contains supplementary material available at 10.1186/s12864-023-09935-9.

## Background

Short tandem repeats (STRs), also known as microsatellites, are DNA sequences composed of either identical (perfect) or highly similar (imperfect) short repetitive units (Supplement Fig. 1) [1]. By definition, the length of the repeated unit is usually shorter than 6bp [2]. STRs are typically flanked by patternless sequences. Since their first characterization in vivo, STRs have been found throughout the genome of both prokaryotes and eukaryotes [3–5]. Under the common definition of STR, more than 3% of human genome reference contains STR sequences, and about 90% of known human genes contain at least one STR locus within the protein-coding regions [2, 6].

STR variants include both nucleotide and length changes, resulting in both mismatches and repeat insertion/deletions (rINDELs). The slippage model first proposed by Kornberg is one widely accepted mechanistic model explaining the high mutation rate at STRs compared to non-STR regions [2, 7, 8]. This model posits that the length of the STR repeat sequence can either expand (increase repeat number) or contract (decrease repeat number) due to a mispairing of the repetitive sequence in the nascent strand to the template strand during DNA replication. This mispairing creates a loop in either the nascent or template strand thus leading to a larger or smaller tandem repeat number in the newly formed DNA strand. In most cases STRs vary by only a single repeat addition or subtraction, but in some cases the STR loci can expand or contract by several thousand repeats [9, 10]. Such length variations may cause structural disruption and result in altered gene expression when they happen within protein coding or non-coding regulatory regions [11–13]. The majority of research into the biological relevance of STRs focuses on the impact of the size of STRs, or the total number of repeated DNA units on each allele at the STR locus [9, 10]. Pathogenic STR expansions cause multiple severe human neurological disorders, including Huntington disease, amyotrophic lateral sclerosis (ALS), fragile X syndrome, and Friedreich ataxia [14–18]. Interestingly, the length of the expansion has been shown to vary in different tissues and cells within the same individual which gives rise to mosaicism [18–20]. In fact, mosaicism has been reported in both clinical cases and mouse models for multiple disease associated STR loci [21–28]. In addition to the contribution of STRs in disease, the high variation rate of STRs also provides polymorphic DNA markers in every individual. Thus, STRs can also be important targets in kinship determination and identity verification when a reliable genotyping method is available [29–31].

The unique properties of STRs make the genotyping of these sites extremely challenging. Historically STRs genotyping was done using repeat-primed polymerase chain reaction (RP-PCR) and southern blotting, however, these approaches are inefficient and require advance knowledge of the target site [32–34]. Genome sequencing technologies offer the potential for a more efficient and more cost-effective way to genotype STRs genome-wide and without bias. Short read sequence outputs have been adopted more widely because application of the emerging long reads sequencing technologies are still limited by cost and high sequencing error rates [35]. Although small STR expansions or contractions can be identified via standard variant calling pipelines as small insertion-deletion variants, the robustness and accuracy of the genotype can be significantly affected by the structural complexity of the STRs, especially when the variant size exceeds the sequenced read lengths [36]. Efforts have been made to develop computational tools specifically for STR realignment and variation calling [37–46], but significant challenges still exist. Many of the STR calling pipelines require the user to provide target STR loci with inflexible input requirements. A recently developed tool ExpansionHunter Denovo does not require information of STR loci and allows for an unbiased screen. ExpansionHunter Denovo uses only paired reads composed of one read mapping to the flanking region and one read mapped to only the region of repeated sequence to detect signals of expansions. This approach only applies to long expansions limiting the ability to genotype specified STR loci when they have no or only small size variations [47]. Furthermore, to our knowledge there are very limited options to detect mosaicism at STR loci which has been observed in some individuals [20]. While the link between somatic mutations and cancer and neurological disorders has been well established, the full contribution of somatic STR variants in disease is yet to be revealed [20, 48, 49]. Given the high mutability of STR variants, post-zygotically acquired pathogenic STR expansions and contractions, which would give rise to mosaicism, may be more involved in disease risk than currently appreciated [25, 28, 50–61].

Here we have developed a novel STR variant calling tool, LUSTR (LU developed STR toolkit), for short read next generation sequencing which offers accurate germline and somatic STR calling in a highly user-friendly format.

## Implementation

**Fig. 1:**
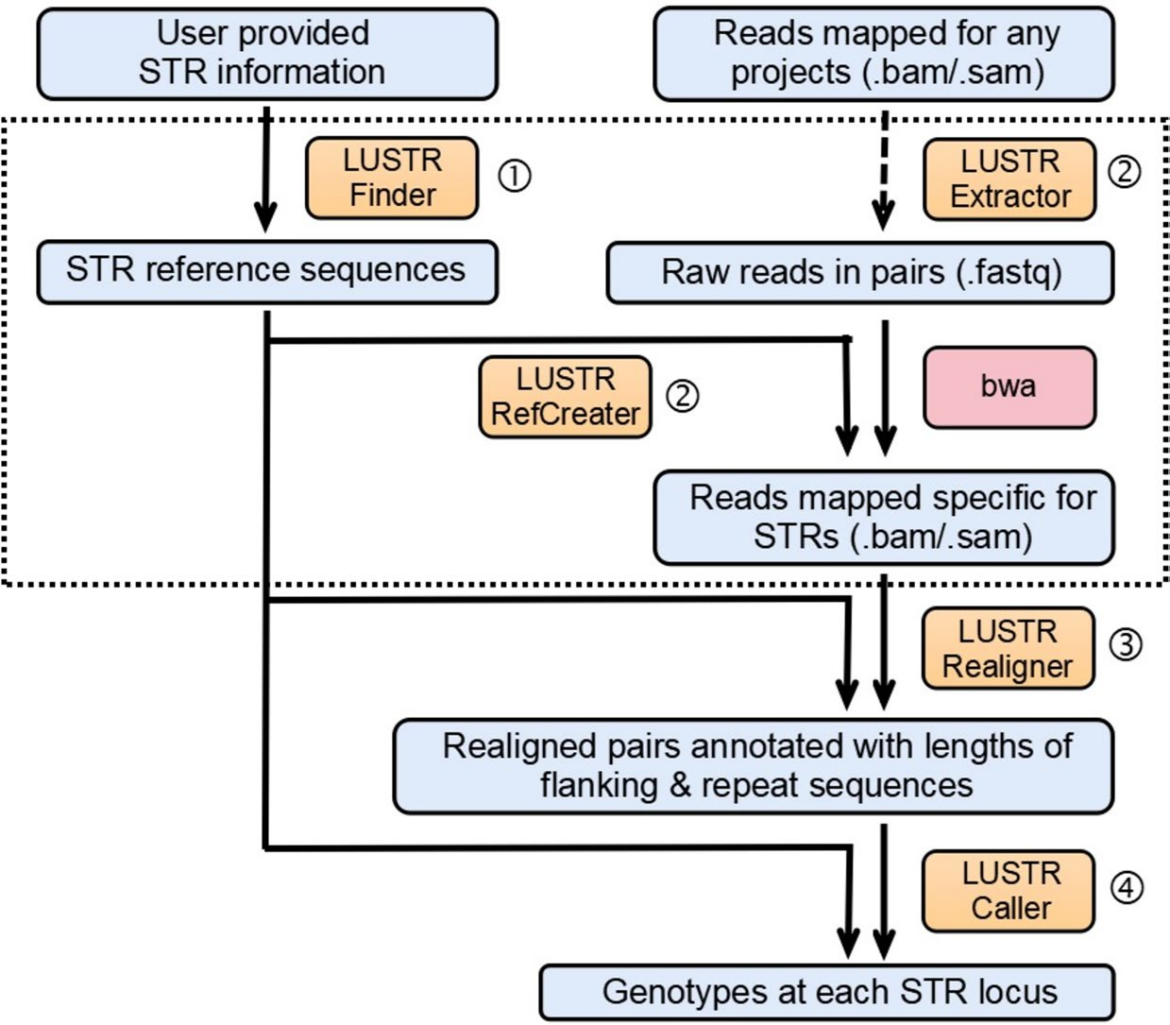
LUSTR pipeline and modules. LUSTR distinguishes itself from other existing pipelines or tools in the following aspects: (1) A “finder” module to standardize extraction of genomic STR regions to be genotyped. The “finder” module aims to simplify the information required to target specific STRs, diminish the impact of imperfect input, and provide flexibility to allow easier user customized target lists, ranging from unbiased compilations for genome-wide scans or a small number of targeted STR sequences. (2) Instead of directly processing mapped reads (.bam files) obtained from alignment pipelines not necessarily optimized for STRs, LUSTR de novo remaps the raw reads (.fastq files) to STR references defined in the “finder” module, with parameters adjusted specifically for STR calling to enhance the performance. We provide an “extractor” module to retrieve reads from the.bam file if raw reads are not available. This remapping step and the “finder” module, indicated by a dashed rectangle, are unique to LUSTR and are not available in other STR calling pipelines. (3) LUSTR implements a flexible two-step strategy for STR genotyping, separating the local realignment step in the “realigner” module to incorporate reads that may have been discarded during the mapping process, and a freestanding calling step in the “caller” module which processes the realignment results to estimate the genotypes for each STR. This modular approach allows for precise tracking of reads through realignment which is critical for debugging and performance evaluation, and allows easier implementation of necessary updates or incorporation of project specific optimization. (4) The “realigner” module applies both flanking-guided and repeat-guided realignment to ensure both accuracy and sensitivity. (5) The “caller” module allows fractional multiallelic STR genotyping results amenable to the calling of germline or somatic expansions or contractions. (6) LUSTR minimizes the prerequisites and only requires pre-installations of *samtools* and *bwa*

We designed the LUSTR pipeline to provide an accurate, robust, and easy-to-use method to call germline and somatic STR variants from short read next-generation sequence data. The pipeline is divided into several modules (Fig. 1), each described below. The Perl scripts used for the LUSTR pipeline are available on GitHub (https://github.com/JLuGithub/LUSTR/releases/tag/STR).

### Finder module

The purpose of this module is to identify the genomic coordinates to extract the repeat and flanking sequences for the STRs the user seeks to genotype. There is no limit to the number of STR sites that can be interrogated. Since the exact sequence of an STR may vary due to the presence of mismatches in some of the repetitive sequences or incomplete repeats (Supplementary Fig. 1), providing exact STR boundaries can be difficult and imprecise. Therefore, in addition to the repeat unit, LUSTR requires only the approximate position of the targeted STR, which can merely include sufficient repeats as seeds to initiate the search. Using this information LUSTR searches the reference sequence for both perfect and imperfect repeats around the given positions, periodically extends the repeats, and automatically determines the boundaries between flanking and repeat sequences using default or user-defined parameters that specify how permissive the user wants to be regarding the extent of mismatch and gaps (Supplementary Fig. 2). The LUSTR-defined genomic coordinates, sequences associated with the targeted STRs, and the parameters used to generate the list will then be carried to the following modules.

### RefCreator module and extractor module

Given the unique requirements for the alignment of sequencing reads at STR loci, LUSTR requires de novo mapping of raw reads to STR loci. Based on the sequences determined by the “Finder” module using the user-defined parameters (Supplementary Fig. 1), the “RefCreator” creates separate references from the flanking and the repeat sequences, as well as artificial references composed by perfect repetitive units of target STRs. In case of unavailability of the original raw reads (.fastq), LUSTR provides the “Extractor” module to pull all of the raw reads from bam files using a single command regardless of the way the bam files are sorted. Alternatively, users can choose *samtools* or other existing tools to prepare the raw reads after the bam files are sorted by reads ID. The mapping of the raw reads to STR references can then be done by existing tools such as *bwa* with appropriate parameters for STRs (defined in the user manual), to provide primary alignments as sam or bam files for the following LUSTR modules. Quality control can be applied either before or after the mapping to reduce false signals in the subsequent steps. Note that this de novo mapping step, as well as the “Finder” module, are unique to LUSTR to increase calling accuracy.

### Realigner module

LUSTR then uses the “Realigner” module to map any unmapped reads and to map the unmapped portions of partially mapped reads from the previous step. Specifically, when the majority of the read is from a flanking sequence, the “Realigner” module will try to align the remaining part to the repeat sequence using the periodic Smith-Waterman algorithm. When the majority of the read is from a repeat sequence, the “Realigner” module will try to align the remaining part to the flanking sequence using the regular Smith-Waterman algorithm. Reads with non-contiguous realignment will be presented as split portions of the read belonging to up-stream flanking, repeat, and down-stream flanking regions of a STR. To analyze each STR in the subsequent step, all realigned reads are categorized according to the STR regions they map to, allowing for single reads to map to multiple different locations if homologous sequences exist. Paired-end reads unable to be mapped to the same target STR(s) are discarded.

### Caller module

In the last step, the “Caller” module collects the information from the alignment procedures described above and lists each potential repeat size at the STR locus that is supported by at least one read. Alleles with repeat sizes short enough to be supported by spanning reads will be determined directly, while the size of long repeats (those exceeding read length) will be estimated by taking the ratio of the number of reads realigned to the flanking and the repeat regions. The quality of the calls can then be determined by inspection of the number of realigned reads and the randomness of their distribution at the STR loci following default or user-provided thresholds. By categorizing pairs supporting each of the potential alleles, the “Caller” module estimates the fraction of each allele, allowing for the possibility of somatic STR variants. Considering the complexity of STRs, the “Caller” module returns the genotyping results in plain text format, which can be easily converted to VCF or other file formats if needed. Furthermore, the “Caller” module also integrates an option to narrow down the STR candidates by generating a list with alleles meeting user-customized thresholds in several features, such as the expansion size, call quality, and allele fraction. Additionally, in the presence of bias detected between upstream and downstream flanking sequences, the “Caller” module will also provides a warning message for users to investigate potential off-targets or complex mutations close by.

## Results

### Application of LUSTR in simulated short reads sequencing datasets

We first tested how well LUSTR performs the local realignment using the “realigner” module, as this step is critical for accurate genotyping and estimating the number of variant alleles present. Simulated reads were generated from the STR locus in human *C9ORF72* gene (Table 1). The *C9ORF72* STR contains tandemly repeated GGGGCC sequences (or GGCCCC on the forward strand), whose expansion is well-studied and known to be associated with ALS (Supplementary Fig. 1). We simulated individual libraries of *C9ORF72* STR alleles with different repeat sizes as follows: (Library 1) allele with the original repeat size (62bp by the default parameters of LUSTR Finder module), (Library 2) expanded allele with 2 times repeats to the original size, (Library 3) expanded allele with 4 times repeats to the original size which exceed standard short read lengths, (Library 4) contracted allele with half number of repeats to the original size, and (Library 5) an allele missing the repeats entirely. Twenty thousand raw reads with lengths of 150 nucleotides were generated in pairs for each library, randomly from the 2X1000bp flanking sequences and the repeat regions. Note that by these settings, the repeat region of *C9ORF72* STR in Library 3 was unable to be fully spanned by any reads due to the length limitation. To simulate sequencing errors, we allowed mismatches, insertions, and deletions (indels) at each nucleotide position at a rate of 0.5%. Raw simulated pairs were then processed following the LUSTR pipeline. The realignment annotations by the LUSTR “realigner” module of flanking and repeat lengths were compared to the records during the generation of the raw reads, and the repeat size estimations by the “caller” module were then compared to the expectation (Table 1). Notably, LUSTR showed high specificity in all libraries and successfully excluded all pairs that were not generated in the forward-reverse pattern (true negative) without calling any positive signals incorrectly (false positive). Among the remaining pairs, LUSTR also exhibited high sensitivity > 99% by successfully retrieving most of the positive pairs (true positive) and missing only a few pairs in certain libraries (false negative). The false negative calls arose because of the mismatches or INDELs that occasionally occurred within correlated reads, which rendered the realignment scores below the threshold and triggered them to be discarded. Moreover, LUSTR annotated > 99% of the true positive pairs identically to the way they were generated, with only a few pairs annotated imperfectly. We found most of the misannotated pairs were due to simulated sequencing errors at the exact boundary between the flanking and repeat regions, which resulted in one nucleotide shifts in the annotation results. These results show that LUSTR was both sensitive and specific to realigning raw reads to the STR loci.

**Table 1:** Performance of LUSTR in genotyping simulated short reads sequencing libraries

Library	Description	Expected	Total	Effective	True Positive (TP)		True Negative	False Positive	False Negative	Estimated Size
		Size (nt)	Pairs	Pairs^a^	Perfect	Imperfect	(TN)	(FP)	(FN)	by LUSTR (nt)
1	Original	62	10000	532	530	2	9468	0	0	62
2	Expansion (2X)	124	10000	665	657	6	9335	0	2	124
3	Expansion (4X)	248	10000	908	905	2	9092	0	1	248—256
4	Contraction (0.5X)	30	10000	452	449	1	9548	0	2	30
5	Knockout	0	10000	388	385	3	9612	0	0	0
	Total		50000	2945	2926 (99.4%)	14 (0.5%)	47055 (100%)	0 (0%)	5 (0.2%)	

Alleles with repeat lengths shorter than read length (150nt) were directly called by LUSTR (libraries 1, 2, 4, and 5), while those beyond the length were estimated by LUSTR as a range by excluding or including pairs containing merely repeat sequences (library 3)

^a^Effective pairs indicate reads overlapping with the repeat sequence and paired by forward-reverse direction

**Fig. 2:**
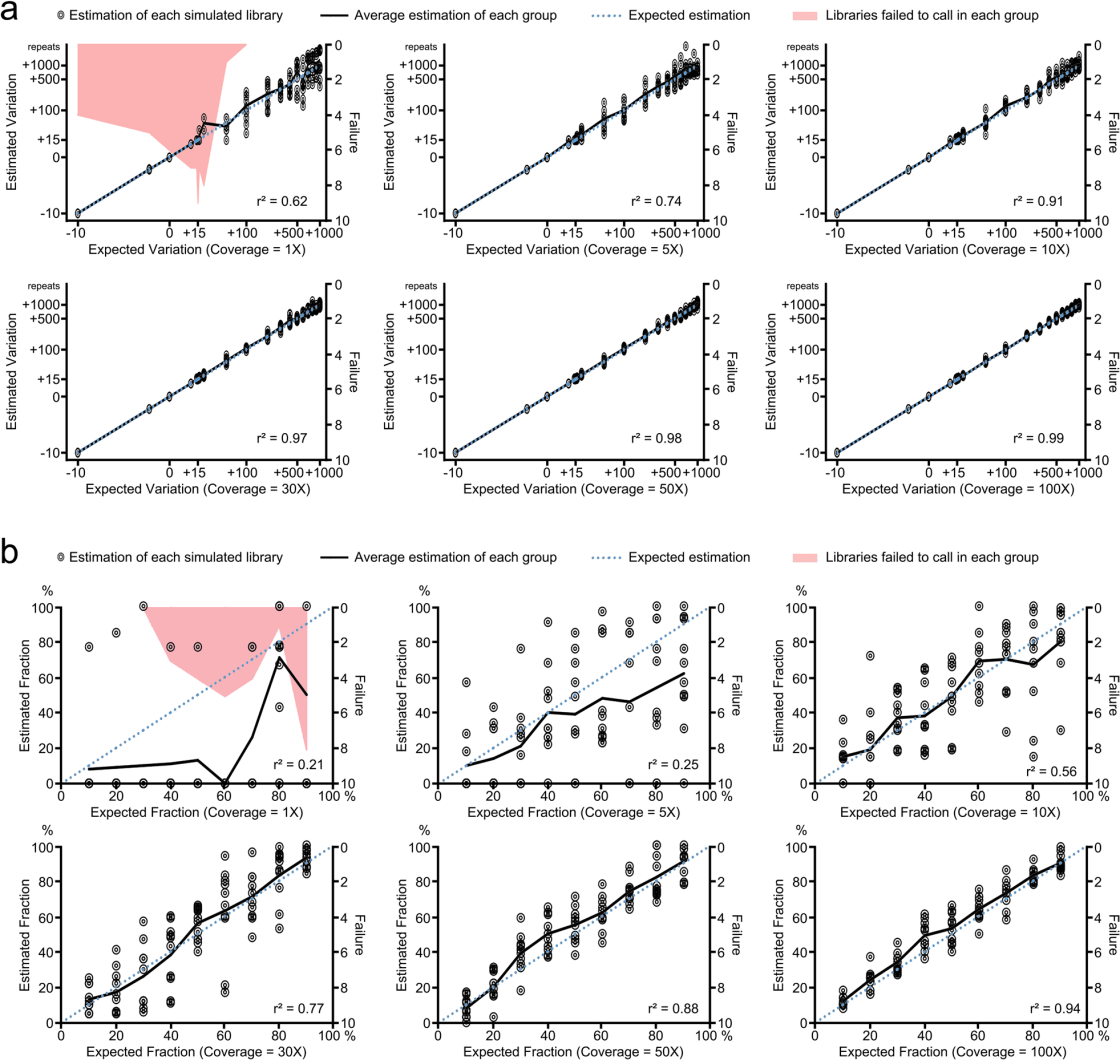
LUSTR is robust in tests with simulated libraries. To test the performance of LUSTR in size and allele fraction estimations, we generated simulated reads from *C9orf72* locus including 2X1000bp flanking regions and the repeats of (**a**) homozygous alleles with different expanded or contracted repeat sizes (ranging from -10.3 to + 1000), and (**b**) heterozygous alleles with one reference allele and one expanded allele (+ 100 repeats), mixed by different fractions. Reads 150 nucleotides in length were generated in pairs with an error rate of 0.5% including mismatches, insertions, and deletions, under different average coverage ranging from 1 to 100X. Each combination was repeated 10 times as a group. The number of failed libraries in each group, which were due to low coverage and mainly for 1X coverage condition, is indicated by red shade. For successfully called libraries, we examined the estimated repeat size variants (**a**) and then estimated the fraction of the reference allele (**b**). The observed and expected are shown for each scenario evaluated. We compared the average result in each group (indicated by a black solid line) with the expectation (indicated by a blue dotted line) and calculated the square of correlation coefficient (r^2^). Among the sizes evaluated, we specifically tested the repeat size variations for the deletion allele (-10.3), reference allele (0), and allele with repeat sizes close to reads length (+ 15) in Fig. 2a. For size estimation (**a**), LUSTR showed robust performance starting from 5X coverage and became very close to the expectations from as low as only 10X coverage. For fraction estimation (**b**) LUSTR required higher coverage, but still exhibited reliable estimates showing the expected allelic ratio with only 10X coverage. This result showed that LUSTR robustly infers both repeat size and allele fraction estimations even for low coverage libraries

We next tested the ability of LUSTR to estimate the size of STR from short reads (Fig. 2a). We simulated homozygous *C9ORF72* STR references with different repeat sizes along with 2X1000bp flankings, and randomly generated forward-reverse 150 nucleotide pairs from each of them. Mismatches or INDELs were allowed at each nucleotide position at a rate of 0.5% to imitate expected sequencing errors. To test the robustness of LUSTR under low sequencing depth, we generated the libraries under different average coverages varying from 1 to 100X. Each condition was repeated 10 times independently, and the raw pairs in each simulated library were processed by LUSTR up through the “caller” module. Individual size variation estimation by LUSTR for each library was shown in Fig. 2a, and the average of each condition was compared to the expectation. We also calculated the square of the correlation coefficient (r^2^) to summarize the ability of LUSTR to call expected sizes under different coverage conditions. LUSTR successfully estimated the STR size variation in libraries with sequencing depth as low as 5X (r^2^ = 0.74), and performed more accurate estimations by the increase of sequencing depth (r^2^ = 0.97 at 30X, Fig. 2a). LUSTR was even able to make an accurate estimation when the STR repeat sizes were close to the simulated read lengths (150bp, variation + 15). These results indicated that LUSTR robustly estimates STR sizes with high accuracy.

The estimation of STR allele fraction has not been explored to any great extent with existing STR calling tools but is essential for somatic variant analysis. Therefore, we further tested the ability of LUSTR to accurately determine STR allele fraction (Fig. 2b). We simulated heterozygous *C9ORF72* STR references composed of two alleles along with 2X1000bp flankings: one with a normal *C9ORF72* STR repeat size (62bp including 18bp perfect repeat units), and the other with a very large expansion in the range commonly found in humans with ALS or FTD (about 100 repeat units longer than reference). The normal *C9ORF72* STR allele fraction was then varied from 10 to 90%. Raw pairs of 150 nucleotides were randomly generated in a forward-reverse pattern under different average coverages varying from 1 to 100X. Randomly generated substitutions or INDELs at a rate of 0.5% were incorporated to account for expected sequencing errors. Simulations for each allelic fraction evaluated were repeated 10 times and independently processed by LUSTR. The estimated allelic fraction of the original *C9ORF72* STR allele in each library is shown in Fig. 2b. The average of each condition was compared to the expectation. We found that the estimation of STR allele fraction required higher sequencing depth compared to that required for non-mosaic STR sizing. Although LUSTR exhibited a correlation between the estimation averages and the expectations starting from 10X coverage (r^2^ = 0.56), it did not return a reliable estimation for individual libraries until 30X (r^2^ = 0.77) or 50X (r^2^ = 0.88) coverage (Fig. 2b). These results indicate that LUSTR is able to successfully estimate the fractions of STR alleles in deep sequenced short reads libraries, although the performance, as expected, could be affected by insufficient realigned reads when sequencing depth was low.

### Identification of known STR variants from publicly-available sequence data using LUSTR

We next tested the ability of LUSTR to correctly identify STR variants in a database with benchmarking variant calls defined by the Genome in a Bottle Consortium (GIAB). GIAB integrates multiple short and linked read sequencing datasets to provide benchmark calls for human genomes and provides a valuable source for the optimization and validation of bioinformatics tools [62]. We downloaded the MGISEQ (150 nucleotide read length) and the BGISEQ (100 nucleotide read length) sequenced pair-ended short reads libraries by their availability for the Ashkenazim trio and the Chinese trio from GIAB. In addition to the analysis for each individual library, we also generated and analyzed merged libraries when the same individual was sequenced multiple times or across multiple sequencing lanes (Tables 2 and 3, Supplementary Table 1). We then selected 13 STR loci that were known to be associated with neurological disorders and thus have been used to validate existing STR calling software [42]. The raw pairs of each merged and individual library were processed by LUSTR using the default settings, and the genotype calls for the listed STR loci were compared with variant calling files (VCFs) provided by GIAB.

**Table 2:** Performance of LUSTR and ExpansionHunter in identifying STR variants reported in the GIAB database (Ashkenazim Trio)

Loci	Father			Mother					Child		
	Call by GIAB	Call by LUSTR (merged)	Call by EH (merged)^a^	Call by GIAB	Call by LUSTR (merged 1)	Call by EH (merged 1)	Call by LUSTR (merged 2)	Call by EH (merged 2)	Call by GIAB	Call by LUSTR (merged)	Call by EH (merged)
ATN1 (CAG) 12:7045880–938	NA	0 (100 ± 0%)	0/0	-7/-2	-7 (46 ± 40%) -2 (54 ± 40%)	-7/-2	-7 (32 ± 22%) -2 (68 ± 22%)	-7/-2	-2/0	-5 (5 ± 16%) -2 (23 ± 17%) 0 (72 ± 17%)	-2/0
ATXN1 (TGC) 6:16327865–955	0/ + 1	0 (74 ± 19%) + 1 (26 ± 19%)	0/ + 1	0/ + 1	-1 (36 ± 23%) + 1 (64 ± 23%)	-1/ + 1	-1 (29 ± 22%) + 1 (71 ± 22%)	-1/ + 1	0/ + 1	0 (38 ± 11%) + 1 (62 ± 11%)	0/ + 1
ATXN2 (GCT) 12:112036754–823	-1/ + 7	-1 (37 ± 28%) + 7 (63 ± 28%)	-1/ + 7	NA	-1 (100 ± 0%)	-1/-1	-1 (95 ± 17%)	-1/-1	NA	-1 (56 ± 25%) + 7 (44 ± 25%)	-1/ + 7
ATXN3 (CTG) 14:92537355–96	NA	+ 6.7 (57 ± 9%) + 9 (43 ± 9%)	+ 7/ + 9	0/ + 13	0 (54 ± 11%) + 13 (46 ± 11%)	0/ + 13	0 (60 ± 9%) + 12.7 (13 ± 10%) + 13 (27 ± 10%)	0/ + 13	NA	+ 9 (61 ± 9%) + 12.7 (19 ± 10%) + 13 (13 ± 10%) + 13.3 (7 ± 10%)	+ 9/ + 13
ATXN7 (GCA) 3:63898361–423	NA	0 (23 ± 39%)	0/ + 1	0/ + 3	+ 3 (100 ± 0%)	0/ + 3	0 (48 ± 52%) + 2.7 (52 ± 52%)	0/ + 3	0/ + 3	0 (23 ± 77%) + 3 (77 ± 77%)	0/ + 3
ATXN10 (ATTCT) 22:46191235–304	0/ + 2	0 (63 ± 8%) + 2 (37 ± 8%)	0/ + 2	-2/-1	-2 (45 ± 10%) -1 (55 ± 10%)	-2/-1	-2 (64 ± 9%) -1 (36 ± 9%)	-2/-1	-2/ + 2	-2 (59 ± 6%) + 2 (41 ± 6%)	-2/ + 2
C9ORF72 (GCCCCG) 9:27573483–544	-1/-1	-1 (100 ± 0%)	-1/-1	-1/-1	-1 (100 ± 0%)	-1/-1	-1 (100 ± 0%)	-1/-1	-1/-1	-1 (100 ± 0%)	-1/-1
CACNA1A (CTG) 19:13318673–712	-2/-1	-2 (77 ± 33%) -1 (23 ± 33%)	-2/-1	NA	-2 (100 ± 0%)	-2/-1	-1 (100 ± 0%)	-2/-1	-2/-2	-2 (100 ± 0%)	-2/-2
CBL (CGG) 11:119077000–33	0/ + 5	0 (47 ± 13%) + 5 (53 ± 13%)	0/ + 5	NA	0 (63 ± 46%)	0/ + 1	0 (93 ± 21%)	0/ + 1	NA	0 (71 ± 9%)	0/0
DMPK (CAG) 19:46273463–524	-9/-7	-9 (52 ± 7%) -7 (48 ± 7%)	-9/-7	NA	-15 (64 ± 9%) -9 (36 ± 9%)	-15/-9	-15 (45 ± 7%) -9 (55 ± 7%)	-15/-9	NA	-9 (100 ± 0%)	-9/-9
HTT (CAG) 4:3076604–67	-2/-2	-2 (100 ± 0%)	-2/0	-2/ + 5	-2 (44 ± 56%) + 5 (56 ± 56%)	-2/ + 5	-2 (70 ± 38%) + 5 (30 ± 38%)	-2/ + 5	-2/ + 5	-9 (4 ± 13%) -2 (45 ± 17%) + 5 (51 ± 17%)	-2/ + 5
JPH3 (GCT) 16:87637889–935	0/ + 2	0 (55 ± 6%) + 2 (45 ± 6%)	0/ + 2	0/ + 2	0 (79 ± 10%) + 2 (21 ± 10%)	0/ + 2	0 (53 ± 7%) + 2 (47 ± 7%)	0/ + 2	+ 2/ + 2	+ 2 (100 ± 0%)	+ 2/ + 2
PPP2R2B (GCT) 5:146258291–322	NA	0 (100 ± 0%)	0/0	NA	0 (100 ± 0%)	0/0	0 (100 ± 0%)	0/0	NA	0 (100 ± 0%)	0/0

*NA* not available (genotype not provided by GIAB in the VCF). Unlike LUSTR, we could not distinguish between reference genotype called or no call provided from GIAB data. To compare LUSTR calls to those from GIAB in these cases, we assigned concordant genotypes when dNA was reported by GIAB and LUSTR supported a reference genotype; we assigned a discordant genotype when GIAB reported NA and LUSTR called an STR variant at that locus. Name, repeat unit, and location in genome (build 37) of each STR are shown in the first column. STR variants expected by GIAB calls or genotyped by LUSTR are shown as repeat number changes compared to reference (build 37). Alleles called by LUSTR are shown by each line, and expansions called by estimation are shown as a range in square brackets. The estimated allele fraction and uncertainty range by LUSTR are shown in parentheses following each correlated genotyped allele. All libraries of the Ashkenazim trio were sequenced by MGISEQ platform. All libraries of the Chinese trio were sequenced by BGISEQ platform except the repeats of the child which were sequenced by MGISEQ. While the supplementary table shows the results of all individual libraries, this table shows the results by merging the two libraries. By default, library 1 and library 2 of each member (Supplementary Table 1) were merged. For the mother in the Ashkenazim trio, we merged library 1 and library 2 as merged 1, and library 3 and library 4 as merged 2

^a^*EH* ExpansionHunter

**Table 3:** Performance of LUSTR and ExpansionHunter in identifying STR variants reported in the GIAB database (Chinese Trio)

Loci	Father			Mother			Child				
	Call by GIAB	Call by LUSTR (merged)	Call by EH (merged)	Call by GIAB	Call by LUSTR (merged)	Call by EH (merged)	Call by GIAB	Call by LUSTR (merged)	Call by EH (merged)	Call by LUSTR (MGI merged)	Call by EH (MGI merged)
ATN1 (CAG) 12:7045880–938	-5/ + 4	-5 (31 ± 23%) + 4 (69 ± 23%)	-5/ + 4	NA	0 (50 ± 12%) + 2 (50 ± 12%)	0/ + 2	NA	0 (26 ± 19%) + 4 (74 ± 19%)	0/ + 4	0 (44 ± 16%) + 4 (56 ± 16%)	0/ + 4
ATXN1 (TGC) 6:16327865–955	NA	-3 (40 ± 60%) -1 (60 ± 60%)	-3/-1	NA	-1 (16 ± 16%) [+ 4.2, + 13.7] (76 ± 25%)	-1/ + 3	NA	-1 (100 ± 0%)	-1/-1	-1 (100 ± 0%)	-1/-1
ATXN2 (GCT) 12:112036754–823	NA	-4 (8 ± 26%) -1 (92 ± 26%)	-1/-1	NA	-1 (100 ± 0%)	-1/-1	NA	-1 (100 ± 0%)	-1/-1	-1 (70 ± 41%)	-1/-1
ATXN3 (CTG) 14:92537355–96	NA	0 (100 ± 0%)	0/0	0/ + 6	0 (59 ± 7%) + 6 (41 ± 7%)	0/ + 6	NA	0 (100 ± 0%)	0/0	0 (100 ± 0%)	0/0
ATXN7 (GCA) 3:63898361–423	NA	0 (100 ± 0%)	0/ + 2	NA	0 (51 ± 36%) + 2 (49 ± 36%)	0/ + 2	0/ + 2	0 (81 ± 51%) + 2 (19 ± 51%)	0/ + 2	0 (84 ± 84%)	+ 2/ + 9
ATXN10 (ATTCT) 22:46191235–304	+ 3/ + 7	+ 2.8 (23 ± 37%) + 3 (37 ± 37%) [+ 7.9, + 9.7] (40 ± 25%)	+ 3/ + 6	NA	-0.2 (34 ± 15%) 0 (66 ± 15%)	0/0	NA	0 (53 ± 24%) + 4.2 (47 ± 24%)	0/ + 6	0 (33 ± 23%) + 7 (67 ± 23%)	0/ + 7
C9ORF72 (GCCCCG) 9:27573483–544	NA	-1 (100 ± 0%)	-1/-1	-1/ + 3	-1 (45 ± 27%) + 3 (54 ± 27%) + 40 (1 ± 5%)	-1/ + 3	-1/-1	-1 (100 ± 0%)	-1/-1	-1 (100 ± 0%)	-1/-1
CACNA1A (CTG) 19:13318673–712	NA	0 (49 ± 19%) + 1 (51 ± 19%)	0/ + 1	NA	0 (100 ± 0%)	0/0	NA	0 (100 ± 0%)	0/0	0 (36 ± 36%)	0/0
CBL (CGG) 11:119077000–33	0/ + 1	0 (67 ± 17%) + 1 (33 ± 17%)	0/ + 1	NA	0 (100 ± 0%)	0/0	NA	0 (100 ± 0%)	0/0	0 (100 ± 0%)	0/ + 1
DMPK (CAG) 19:46273463–524	-5/-4	-5 (56 ± 15%) -4 (44 ± 15%)	-5/-4	NA	-7 (100 ± 0%)	-7/-7	-7/-4	-7 (43 ± 9%) -4 (57 ± 9%)	-7/-4	-7 (53 ± 8%) -4 (47 ± 8%)	-7/-4
HTT (CAG) 4:3076604–67	-2/-1	-2 (17 ± 27%) -1 (83 ± 27%)	-2/-1	-2/-1	-2 (29 ± 23%) -1 (71 ± 23%)	-2/-1	-1/-1	-1 (96 ± 14%) 0 (4 ± 14%)	-1/-1	-1 (100 ± 0%)	-1/-1
JPH3 (GCT) 16:87637889–935	-1/ + 2	-1 (42 ± 12%) + 2 (58 ± 12%)	-1/ + 2	NA	0 (100 ± 0%)	0/0	0/ + 2	0 (29 ± 9%) + 2 (71 ± 9%)	0/ + 2	0 (37 ± 5%) + 2 (63 ± 5%)	0/ + 2
PPP2R2B (GCT) 5:146258291–322	NA	+ 6 (55 ± 13%) + 8 (45 ± 13%)	+ 6/ + 8	NA	+ 3 (46 ± 14%) + 6 (54 ± 14%)	+ 3/ + 6	+ 3/ + 6	+ 3 (62 ± 11%) + 6 (38 ± 11%)	+ 3/ + 6	+ 3 (44 ± 7%) + 6 (56 ± 7%)	+ 3/ + 6

See legend for Table 2

^a^*EH* ExpansionHunter

Across all the Ashkenazim and Chinese trio libraries and the 13 loci, there were a total of 54 opportunities to compare the genotype provided by GIAB to that called by LUSTR (Tables 2 and 3). For 48 out of the 54 comparisons (88.9%), the predominant allele(s) identified by LUSTR matched that of the benchmark GIAB calls. Among the concordant calls, LUSTR also detected two contracted STR variants at the *ATN1* and *HTT* loci for the son of the Ashkenazim trio at low levels, one with a five repeat units contraction by 5% allele fraction and the other with a nine repeat units contraction by 4% allele fraction (indicated as -5 and -9 in Tables 2 and 3, respectively). Although these small fraction alleles were not called by GIAB, they were supported by some reads realigned to the loci (Supplementary Fig. 4). This could be due to sequencing errors that generated a small fraction of reads artificially revealing the variants, or it could indicate the real presence of somatic STR variants at these loci. A minor fraction of reads supporting an allele that was + 12.7 were also detected at the *ATXN3* loci in the Ashkenazim trio compared to the expected + 13. This minor discrepancy was attributed to likely by sequencing errors or slight interpretation differences. For the six discordant calls (11.1%), they were either small differences in repeat count unlikely to alter the interpretation (i.e., for the Ashkenazim trio LUSTR called -1/ + 1 repeats for the two *ATXN1* alleles whereas GIAB reported 0/ + 1) or due to reads supporting variant alleles being absent in specific libraries (i.e., *ATXN7* in the mother of the Ashkenazim trio and in the child of the Chinese trio) (Supplementary Table 1a and b and Supplementary Fig. 3).

For the 50 instances without GIAB calls (NAs), LUSTR called 34 genotypes identical or close to the reference (68%), which likely explains the absence of calls in GIAB VCFs. In addition to observing a high rate of calling concordance, there were several cases where LUSTR detected a genotype that was not called by GIAB. For example, at the DMPK locus, LUSTR called a genotype of -15, -9 in two sequencing runs for the mother of the Ashkenazim trio that was not reported by GIAB (Tables 2 and 3). The reason this was not called in GIAB is unclear. However, in all cases, there was clear sequence read evidence supporting the presence of these alleles (Supplementary Fig. 5). The LUSTR genotype calls in the child also followed a Mendelian inheritance pattern which further supports the accuracy of the calling (Tables 2 and 3).

Since ExpansionHunter provides curated information and input format for these 13 STR loci, we also ran ExpansionHunter (ver 4.0, default settings) and compared the results in all of the eight merged libraries to further evaluate the performance of LUSTR. Among all the 104 comparisons from both Ashkenazim and Chinese trios, LUSTR showed 94 calls (90.4%) identical or equivalent to ExpansionHunter results, including those loci referred above where LUSTR called genotypes significantly different from GIAB database (Tables 2 and 3, Supplementary Fig. 5). Among the 10 calls that were discordant between LUSTR and ExpansionHunter, there were instances where ExpansionHunter was able to reveal the hidden allele missed by LUSTR (e.g. *ATXN7* in the mother from Ashkenazim trio), but also instances where LUSTR showed more convincing results by raw reads inspection (e.g. *HTT* in the father from Ashkenazim trio) (Tables 2 and 3). These results collectively support that LUSTR can accurately genotype STR variant alleles in short reads sequencing libraries.

### LUSTR was accurate and robust to call mosaic STR variants in the in silico mixture libraries

We next tested the ability of LUSTR to call mosaic STR variants by mixing short reads from real data libraries in silico. We selected two MGISEQ sequenced libraries with equal sequencing lengths, one from the father of the Ashkenazim trio and the other from the child of the Chinese trio. We generated an in silico mixture by randomly selecting varying proportions of reads (Table 4) from the two libraries. The mixed libraries were then processed by LUSTR for the 13 tested STR loci shown in Tables 2 and 3. To validate the performance, we first assumed the STR genotypes of the two samples by integrating both GIAB calls and reliable LUSTR calls in the previous tests. We then estimated the expected STR allele fractions in the mixture libraries by assuming that both original samples had homozygous or heterozygous germline genotypes at these loci (i.e., 100% or 50% variant allele frequency, Table 4). The expected calls were then compared with the calls by LUSTR. In the mixed library consisting of a 1:2 ratio of the two genomes, LUSTR successfully called the alleles with fractions very close (< 10%) to expected for six out of the 13 STRs (*ATN1*, *ATXN3*, *C9ORF72*, *CBL*, *DMPK*, and *HTT*) (46.2%, Table 4). For 5 STRs (*ATXN2*, *ATXN10*, *CACNA1A*, *JPH3*, and *PPP2R2B*), LUSTR called allelic fractions deviating greater than 10% from expectation. This could be due to read bias in the original samples or sampling error (Table 4, Supplementary Table 1a and b). LUSTR missed STR alleles for *ATXN1* and *ATXN7*, but these were due to missing or low-quality reads supporting the non-dominant alleles in the original libraries (Table 4, Supplementary Table 1).

**Table 4:** Ability of LUSTR to estimate allele fraction by in silico mixture of samples

Loci	Original Genotype^a^		Expectation	LUSTR estimated allelic fraction			
	Father (32.8% of mixture) Ashkenazim Trio library 1	Son (67.2% of mixture) Chinese Trio MGI library 1		(520837211 pair-ends)			
ATN1 (CAG) 12:7045880-938	0/0	0/+4	0 (67%) +4 (33%)	0 (74 ± 26%) + 4 (26 ± 26%)			^***^
ATXN1 (TGC) 6:16327865-955	0/+1	-1/-1	-1 (67%) 0 (17%) +1 (17%)	-1 (57 ± 26%) 0 (43 ± 26%)			-
ATXN2 (GCT) 12:112036754-823	-1/+7	-1/-1	-1 (83%) +7 (17%)	-1 (61 ± 48%) + 7 (21 ± 51%)			^*^
ATXN3 (CTG) 14:92537355-96	+6.7/+9	0/0	0 (67%) + 6.7 (17%) +9 (17%)	0 (68 ± 11%) + 6.7 (21 ± 11%) + 9 (11 ± 11%)			^***^
ATXN7 (GCA) 3:63898361-423	0/0	0/+2	0 (67%) +2 (33%)	0 (100 ± 0%)			-
ATXN10 (ATTCT) 22:46191235-304	0/+2	0/+7	0 (50%) +2 (17%) +7 (33%)	0 (40 ± 20%) + 2 (36 ± 20%) + 7 (24 ± 24%)			^*^
C9ORF72 (GCCCCG) 9:27573483-544	-1/-1	-1/-1	-1 (100%)	-1 (100 ± 0%)			^***^
CACNA1A (CTG) 19:13318673-712	-2/-1	0/0	-2 (17%) -1 (17%) 0 (67%)	-2 (32 ± 48%) -1 (33 ± 47%) 0 (34 ± 47%)			^*^
CBL (CGG) 11:119077000-33	0/+5	0/0	0 (83%) + 5 (17%)	0 (76 ± 24%) + 5 (24 ± 24%)			^***^
DMPK (CAG) 19:46273463-524	-9/-7	-7/-4	-9 (17%) -7 (50%) -4 (33%)	-9 (17 ± 9%) -7 (51 ± 10%) -4 (32 ± 10%)			^***^
HTT (CAG) 4:3076604-67	-2/-2	-1/-1	-2 (33%) -1 (67%)	-2 (30 ± 23%) -1 (70 ± 23%)			^***^
JPH3 (GCT) 16:87637889-935	0/+2	0/ + 2	0 (50%) + 2 (50%)	0 (35 ± 8%) + 2 (65 ± 8%)			^*^
PPP2R2B (GCT) 5:146258291-322	0/0	+ 3/ + 6	0 (33%) + 3 (33%) + 6 (33%)	0 (17 ± 10%) + 3 (40 ± 11%) + 6 (43 ± 12%)			^*^
Loci	Original Genotype^a^		Expectation	LUSTR estimated allelic fraction			
	Father (8.9% of mixture) Ashkenazim Trio library 1	Son (91.1% of mixture) Chinese Trio MGI library 1		Mixture 1 (384221009 pair-ends)	Mixture 2 (384225464 pair-ends)	Mixture 3 (384223036 pair-ends)	
ATN1 (CAG) 12:7045880-938	0/0	0/ + 4	0 (55%) + 4 (45%)	0 (63 ± 37%) + 4 (37 ± 37%)	0 (67 ± 33%) + 4 (33 ± 33%)	0 (63 ± 37%) + 4 (37 ± 37%)	^***^
ATXN1 (TGC) 6:16327865-955	0/ + 1	-1/-1	-1 (90%) 0 (5%) + 1 (5%)	-1 (100 ± 0%)	-1 (77 ± 34%) 0 (23±34%) (lq)	-1 (77 ± 34%) 0 (23 ± 34%)	-
ATXN2 (GCT) 12:112036754-823	-1/ + 7	-1/-1	-1 (95%) + 7 (5%)	-1 (78 ± 52%)	-1 (78 ± 52%)	-1 (73 ± 73%)	-
ATXN3 (CTG) 14:92537355-96	+ 6.7/ + 9	0/0	0 (90%) + 6.7 (5%) + 9 (5%)	0 (86 ± 13%) + 6.7 (9 ± 13%) + 9 (5 ± 14%)	0 (90 ± 14%) + 7 (5 ± 13%) + 9 (5 ± 14%)	0 (90 ± 14%) +6.7 (5±13%) (lq) + 9 (5 ± 14%)	^***^
ATXN7 (GCA) 3:63898361-423	0/0	0/ + 2	0 (55%) + 2 (45%)	0 (100 ± 0%)	0 (100 ± 0%)	0 (100 ± 0%)	-
ATXN10 (ATTCT) 22:46191235-304	0/ + 2	0/ + 7	0 (50%) + 2 (5%) + 7 (45%)	0 (53 ± 47%) + 7 (47 ± 47%)	0 (53 ± 47%) + 7 (47 ± 47%)	0 (60 ± 40%) + 7 (40 ± 40%)	-
C9ORF72 (GCCCCG) 9:27573483-544	-1/-1	-1/-1	-1 (100%)	-1 (100 ± 0%)	-1 (100 ± 0%)	-1 (100 ± 0%)	^***^
CACNA1A (CTG) 19:13318673-712	-2/-1	0/0	-2 (5%) -1 (5%) 0 (90%)	0 (100 ± 0%)	-2 (49 ± 51%) 0 (51 ± 51%)	0 (100 ± 0%)	-
CBL (CGG) 11:119077000-33	0/ + 5	0/0	0 (95%) + 5 (5%)	0 (87 ± 35%) + 5 (13 ± 35%)	0 (87 ± 35%) + 5 (13 ± 35%)	0 (89 ± 32%) + 5 (11 ± 32%)	^***^
DMPK (CAG) 19:46273463-524	-9/-7	-7/-4	-9 (5%) -7 (50%) -4 (45%)	-9 (11 ± 11%) -7 (49 ± 12%) -4 (41 ± 12%)	-7 (54 ± 14%) -4 (46 ± 14%)	-7 (58 ± 12%) -4 (42 ± 12%)	^***^
HTT (CAG) 4:3076604-67	-2/-2	-1/-1	-2 (10%) -1 (90%)	-1 (100 ± 0%)	-1 (100 ± 0%)	-2 (10 ± 29%) (lq) -1 (90 ± 29%)	^*^
JPH3 (GCT) 16:87637889-935	0/ + 2	0/+2	0 (50%) + 2 (50%)	0 (39 ± 10%) + 2 (61 ± 10%)	0 (33 ± 10%) + 2 (67 ± 10%)	0 (30 ± 10%) + 2 (70 ± 10%)	^*^
PPP2R2B (GCT) 5:146258291-322	0/0	+3/+6	0 (10%) + 3 (45%) + 6 (45%)	0 (7 ± 12%) + 3 (44 ± 13%) + 6 (48 ± 13%)	0 (11 ± 11%) + 3 (43 ± 12%) + 6 (47 ± 13%)	0 (4 ± 12%) + 3 (46 ± 13%) + 6 (50 ± 13%)	^***^

Two in silico mixture sets applying different sample proportions were processed and tested by LUSTR. Mixing was repeated three runs to ensure that non-dominant reads source were represented in the second test for extreme low fraction alleles. Libraries were picked from the father in Ashkenazim trio and the son in Chinese trio, both sequenced by MGISEQ platform (Supplementary Table 1). The list of tested STRs was identical to the one used in the test for GIAB libraries (Tables 2 and 3 and Supplementary Table 1). Name, repeat unit, and location in genome (build 37) of each STR are shown in the first column. The expectations of STR alleles and their fractions in the in silico mixtures (column 4) were calculated based on the original genotypes of the samples and their mixing proportions (column 2 and 3). The estimation results by LUSTR were indicated in either *** (matching expectation), * (mismatching fraction estimation, > 10%) or—(loss of allele). Alleles called but deemed low quality by LUSTR were marked with “lq”. Low quality calls are likely due to low representation of minor alleles in the mixture

^a^The original genotypes of the two samples were determined by both GIAB calls and LUSTR estimations (Supplementary Table 1), with assumption of germline patterns as either homozygous or heterozygous (i.e., no mosaicism at targeted loci)

To further test the ability of LUSTR under more extreme conditions, we then mixed the two samples by an approximate ratio of 1:10 to mimic mosaic STR alleles with fractions as low as 5 or 10% (Table 4). Considering that such low fractions were made by selecting only a few reads from the sample, we performed three replicates to reduce the impact of sampling error that can occur during the mixing (Table 4). LUSTR successfully called the alleles with expected fractions in at least one of the three replicates for 6 out of the 13 STRs (*ATN1*, *ATXN3*, *C9ORF72*, *CBL*, *DMPK*, and *PPP2R2B*) (46.2%, Table 4). Notably, LUSTR was able to call the minor alleles with low fractions (5 or 10%) for *ATXN3*, *CBL*, *DMPK*, and *PPP2R2B* with very close estimations (< 10%) (Table 4). At the *HTT* locus, LUSTR called the correct fraction in one of three mixtures but flagged the call as not being reliable. This suggests that allowing more permissive calling may be needed to capture mosaic STRs. At the *JPH3* locus, LUSTR estimated allelic fractions that did not align well with expectation (> 10% difference) (Table 4). The reason for this is unclear but is likely due to allelic bias from the Chinese trio Son library as shown by original LUSTR calls (Supplementary Table 1a and b). LUSTR consistently missed the minor alleles for *ATXN1*, *ATXN2*, *ATXN7*, *ATXN10*, and *CACNA1A* (Table 4) due to the loss of all reads supporting that allele when randomly sampling from the non-dominant genome.

While noisier than germline calling, these results collectively support the ability of LUSTR to accurately call mosaic STR variant alleles with variant allele fractions as low as 5%. We note however that the accuracy will be greatly influenced by read depth at the locus, as is the case for calling of any allele with low representation in a genome.

### Identification of undiagnosed STR expansions in subjects by unbiased whole genome scan using LUSTR

We next tested the ability of LUSTR to identify clinically significant STR expansions using an unbiased whole genome scan in samples harboring known pathogenic STRs. We collected raw whole genome sequence data (short read paired end sequencing) from three individuals with presumed genetic disorders sequenced as part of the Undiagnosed Disease Network (UDN). These subjects were genetically undiagnosed, but all had STR expansion variants that may explain their phenotype (Table 5). We were blind to the specific phenotypes or genotypes while performing the scans so not to bias the analyses. Two libraries were sequenced for subject 1 and subject 2, and four libraries were sequenced for subject 3 (Table 5). We also collected the libraries from the unaffected parents and siblings for subject 1 and subject 2 to determine inheritance (Table 5). To prepare for the whole genome scan, we used Tandem Repeats Finder [37] to obtain the basic information of STRs across the whole human genome reference (build 37). We ran Tandem Repeats Finder using the recommended settings (match/mis/gap/PM/PI/minscore = 2/-5/-7/80/10/50), selected those STRs located within 1000 bp distance to known genes as defined by UCSC genome annotation database (https://hgdownload.soe.ucsc.edu/goldenPath/hg19/database/), and retrieved a customized list of 162,840 STRs. We then applied the LUSTR “Finder” module to retrieve the standardized STR sequences for these 162,840 loci by default settings (match/mis/gap/stop =  + 2/-5/-7/-30) and generated reference sequences by using the “RefCreater” module.

**Table 5:** Unbiased whole genome scan by LUSTR for known STR expansions in undiagnosed subjects

Subject	Candidate	Number of	STR Variant Candidates	Shared^c^	Non-primary Candidates			Primary	Availability of Relatives^e^	
	Variant^a^	Libraries	number in each library^b^		Likely Non-specific^d^	Medium Call Quality	Total	Candidates	Parents	Siblings
1	GLS	2	258, 345	86	49	30	66	20	both	brother
2	RFC1	2	337, 287	78	44	22	57	21	both	sister
3	RFC1	4	328, 207, 133, 260	33	28	9	32	1	NA	NA

Three undiagnosed subjects were processed blindly by LUSTR for an unbiased whole genome scan. As described, each library was scanned for 162,840 STR loci, and candidate expansions that passed the filtration and shared by libraries of the same subject were further categorized as non-primary or primary candidates, based on their calling qualities and calls in other subjects. Primary candidates were focused on for further evaluation by their details (Table 6)

^a^STR variants identified by the UDN as possibly contributing to the individual's phenotype. LUSTR was run blindly without knowledge of these candidate STR expansions

^b^Number of STR variants that were: (1) expanded, (2) size change ≧100bp, (3) allele fraction ≧5%, (4) realigned pairs ≧15 w/o repeat-only pairs, (5) medium or high calling quality

^c^Number of STR expansions that were called and passed the threshold setting in both libraries for subject 1 or 2, or in at least three libraries for subject 3

^d^Number of STR expansion candidates that were also called in other two subjects

^e^The availability of  sequence data from unaffected parents and siblings

Raw read libraries of the three subjects were mapped to the references generated by LUSTR using *bwa mem*. All bam files from each individual library as well as merged bam files for each subject were then processed blindly by the LUSTR “Realigner” and “Caller” modules against the customized STR list. The parallel processing function provided by LUSTR was applied to reduce the processing time for calling. We set thresholds for the “Caller” module to call all STR loci with alleles expanded larger than 100 bp compared to the human genome reference, allelic fractions larger than 5%, and variant sites called by more than 15 realigned pairs without repeat-only pairs in at least medium calling quality determined by LUSTR “Caller” module. Note that such settings can be relaxed to reduce the risk of false negatives and to capture mosaicism. The STR expansions fulfilling the quality control metrics were then checked to assess whether they were detected in both individual libraries of subject 1 or subject 2, or were detected in at least three individual libraries of subject 3. Following these steps, we identified 86 candidate STR expansions for subject 1, 78 candidates for subject 2, and 33 candidates for subject 3 (Table 5).

Among the 86 candidates for subject 1, 49 STR expansions were also detected with similar or larger sizes in subjects 2 and 3 and assumed to be either benign polymorphisms or sequencing artifacts. Among the 37 remaining we focused on the 20 candidates with high calling quality for primary investigation (Tables 5 and 6). We next looked into the detailed features of these 20 candidates to decide their priority ranking based on the likelihood they may contribute to the individual’s phenotype. Distinct from the previously excluded 49 candidates that passed the threshold and were also called in subjects 2 and 3, many of these 20 candidates were either called in only one of subjects 2 or 3, or called with a smaller expansion or a low allele fraction that didn’t pass the threshold for subjects 2 and 3. This may indicate non-specificity, but could also indicate potential genetic penetrance. We decided to keep them on the list, but took this into consideration when making priority determination (Table 6). Another important feature being considered for the 20 candidates was the reference size of each candidate STR, since the estimation for STR expansions with reference sizes longer than read length was more likely to be affected by sequencing randomness and off-target repeats, compared to those with relatively shorter sizes (Table 6). We also investigated other features such as the locations of the candidates to the affected genes, the potential for off-target alignment or the presence of mutations in the flanking sequences, and the number of called alleles which could indicate complex situations requiring further examination (Table 6). Among all these candidates, the STR expansion at the *GLS* gene, a known pathogenic STR, was deemed the most likely candidate in subject 1 (Table 6). We also identified STR expansions at *ARHGAP28* and other loci with high priorities that may also be worthy of further consideration (Table 6). Once unblinded, we found that the *GLS* expansion was indeed the suspected pathogenic variant identified for subject 1.

**Table 6:** Evaluation of candidate STR expansions by LUSTR unbiased whole genome scan for subject 1

STR	Affected	Region	Distance to	Reference	LUSTR Warning^a^	Specificity^b^		Other Information for Evaluation	Priority^c^
	Gene		nearest exon	Repeat Size		Subject 2	Subject 3		
1	ATAD3C	intergenic	300bp	long	-	smaller	smaller	-	○
2	abparts/IGL@	intron	far away	about reads length	-	-	-	-	○
3	KCHN7	intron	far away	about reads length	-	smaller	smaller	-	-
4	GLS	exon	0bp	short	-	-	-	also called in Father & Brother	●●●
5	IQCB1	intron	2kb	short	5’ flanking mutant	also called	-	also called in Father & Brother	●○
6	SLAIN2	intron	3kb	short	3’ flanking mutant	low %	also called	multiallelic calls	○
7	LOC100507602	intron	far away	about reads length	-	-	-	multiallelic calls	-
8	NSMCE2	intron	far away	about reads length	-	smaller	smaller	-	-
9	PTPRE	intron	600bp	long	-	smaller	smaller	-	-
10	TRPC2	intron	2kb	long	-	smaller	also called	-	-
11	ABCC4	intron	far away	long	-	smaller	smaller	-	-
12	ATG2B	intron	2kb	short	-	low quality	smaller	-	●○
13	TNRC6A	intron	5kb	short	-	smaller	smaller	also called but smaller in Father & Brother	●
14	FA2H	intron	2kb	short	-	also called	-	also in Father, low quality in Mother & Brother	●○
15	CALCOCO2	intron	2kb	short	-	-	smaller	-	●○
16	RNF213	exon	0bp	short	-	smaller	smaller	-	●●
17	ARHGAP28	intron	300bp	short	-	-	low quality	also called in Mother	●●○
18	SIGLEC5	intron	200bp	long	-	smaller	smaller	-	○
19	GYG2	intron	2kb	long	-	smaller	smaller	-	-
20	CHM	intron	20bp	short	-	also called	smaller	-	●○

Details of the 20 primary STR expansion candidates for subject 1 in the unbiased whole genome scan by LUSTR (Table 5)

^a^Warning messages given by LUSTR indicating potential homologous flanking sequences or mutations within flanking regions close to the repeats

^b^Indicating whether the expansion was also called in other subjects. The calls in other subjects did not trigger previous filtration because they were: (1) not called by both other subjects; (2) called by low qualities; or (3) called by low fractions or smaller size variations that didn’t pass the threshold settings

^c^The priority of each candidate STR expansion was determined based on the information collected in this table. The number of ● and ○ indicates the level of priority, where ○ indicate a lower priority contribution compared to ●. Note that this priority did not necessarily mean true or false positive, but rather served as a guidance for further evaluation and confirmation based on raw reads inspection, targeted sequencing, and potential clinical importance

We applied a similar procedure to subjects 2 and 3 and narrowed down the candidate list to 21 and one high quality STR calls, respectively (Supplementary Table 2). However, we could only deem TCF4 STR expansion as a possible candidate for subject 2 and no possible candidates were identified for subject 3. Following unblinding the cases, both harbored likely pathogenic *RFC1* STRs. The *RFC1* STR variants in the two subjects included a replacement of the repetitive “AAAAG” with “AAGGG”, a 1-bp shift, and the expansion (AA + AAAAG × 11 + AAAAAG—> AAA + AAGGG × 10 + AAGAAAAAG—> AAA + AAGGG x n + AAGAAAAAG). This explains why LUSTR, when searching for “AAAAG” repeats under the default settings, actually gave expansion signals at *RFC1* locus for the two subjects by very low realignment coverage and low calling quality, which did not happen for the parents and sibling (Supplementary Table 3). To evaluate the flexibility of LUSTR to fulfill the detection of this complex *RFC1* expansion, we first tried reducing the mismatching penalty. More pairs were realigned, but the calling qualities were not improved adequately for successful detection as merely penalty change did not benefit retrieving repeat dominant reads (Supplementary Table 3). However, by applying a customized alternative *RFC1* STR reference with “AAGGG” repeats accordingly, the *RFC1* expansions were successfully detected with high coverage and quality for both subjects 2 and 3 (Supplementary Table 3). Moreover, by combining both results by the two *RFC1* STR references, LUSTR genotyped an “AAGGG” expansion allele in subject 1 inherited from the mother, as well as four individuals carrying “AAAAG” expansion alleles in the families of subject 1 and subject 2 (Supplementary Fig. 6). These cases exemplify the challenges of STR calling but also demonstrate the flexibility of LUSTR for customization upon user-specified settings. Developing LUSTR to call non-reference STRs sequences de novo is an area for future development of the software.

## Discussion

Besides the utility of STRs in kinship determination and identity verification, STRs have attracted significant attention for their role in human neurological disorders. Genome-wide sequencing offers tremendous potential to identify STRs that may contribute to disease. Despite the recent progress made in calling STR variants in short read sequence data, there is an on-going need for improvements to make calling more user friendly and interpretable [20, 35, 63].

The LUSTR pipeline described here builds on the advantages of several different existing STR variant calling tools [37–45]. LUSTR specifically aims to provide an alternative choice to benefit users with varied conditions or in need of more flexible input requirements (Supplementary Table 4). LUSTR applies the strategy to realign as many reads as possible to each STR locus in order to allow for the most sensitivity and accurate STR calling as possible. It also enables the detection of deviations in allele frequencies that may indicate mosaicism, which has hardly been addressed to date in existing STR callers  (Supplementary Table 4). LUSTR follows the classic pipeline of mapping, local realignment, and then STR calling. However, distinct from other existing tools, LUSTR requires a de novo mapping from the raw reads to STR specific references generated in the pipeline, rather than directly processing bams from whole genome mapping. Although it may increase the cost of running time and storage space, this design aims to improve the sensitivity to specifically call STRs, and reflects the idea that STR mutation should be considered as a unique type of variation that requires a distinctive pipeline from that designed to call SNVs and INDELs. In our tests running LUSTR along with the existing STR variant calling tools, LUSTR and ExpansionHunter showed consistent calls in most cases (> 90%, Tables 2 and 3). For the discordant loci, neither LUSTR nor ExpansionHunter showed a significant overall advantage over the other, indicating that each tool has pros and cons under different conditions. As for the running speed, a single process for a whole genome STR genotyping by LUSTR takes days to finish, varying within about a seven day range depending on several factors including sequencing depth, list size of target STRs, and running platform conditions. This running speed, mostly dictated by the Realigner module, is slower than ExpansionHunter or GangSTR when simple target inputs are supplied, but comparable when off-target information is provided [42, 45]. Moreover, LUSTR allows for parallel processing, which will greatly increase the running speed (Supplementary Table 4). In the local realignment step, LUSTR uses the periodic Smith-Waterman algorithm to solve the challenges of imperfect repeats and sequencing errors that happen within STR repetitive regions. While this approach increases sensitivity for long expansions with an expected trade-off in specificity, we note that parameters in the Finder module and subsequent calling step can be altered to favor specificity over sensitivity. New optional modules are under development to further reduce noise and enhance specificity to benefit certain situations such as cohort-level association analyses.

Long read sequencing technologies that have recently emerged will likely improve STR variant calling. LUSTR is designed based on short read sequenced data which remains much more commonly used due to cost and accuracy limitations of current long reads sequencing technologies. Even when long-read sequencing is more economical and accurate, there will still be large numbers of genomes sequenced with short-read sequencing genomes for which short-read STR variant callers will be still be needed. Newer tools have been developed to incorporate algorithms compatible to long sequenced reads to address this emerging need [46]. Another future development of LUSTR will be focused on ensuring compatibility of the caller with long read sequencing data.

Both the local realignment and the variant calling steps are widely acknowledged as critical factors required for accurate STR variant calling [38–46]. However, the importance of STR sequence definition is often underestimated when STR target list customization is required, which is another important feature where LUSTR will provide an improved experience compared to other existing tools (Supplementary Table 4). The repeat regions of STRs often contain partial or imperfect repetitive sequences, natural SNVs and short INDELs, as well as sequencing errors during the establishment of the reference. Therefore, the boundaries of STRs may vary largely according to different definition rules, making it difficult for users to precisely define STRs regions of the genome. Furthermore, the inconsistent rules applied to STR boundary definition and local realignment may lead to aberrant calls. One solution is to provide an STR list with the optimal format [42, 64]. Although the list can be updated and expanded following newly emerging clinical discoveries, the feature would limit the ability of the user to add new STR loci of interest or that arise for new releases of the reference genome. Beyond the widely used GRCh37/38 genomes, there have been several new genome references, such as Telomere-to-Telomere genome reference (T2T), Han Chinese genome reference (HG00514), and Japanese genome reference (JG2) [65–68]. To apply STR analysis using these novel genomes or even non-human references, users need to be able to easily switch between genomes and add novel STR loci of interest. LUSTR fulfills this need with the Finder module that allows for flexible input and a standardized approach for determining STR boundaries. By automatically applying an exact set of parameters in the following local realignment, it allows easy customization of STR lists and also makes it possible to apply unbiased whole genome-wide scans for STR variants.

STR analysis can also be challenging when certain loci share homologous flanking sequences with other STRs with identical or similar repetitive units. These loci can result in off-target mapping and ultimately inaccurate STR calling. To solve this, LUSTR provides warning messages to indicate signals for potential off-targets or complex mutations close to the STR boundaries that may affect on-target mapping. By flagging these sites, users can investigate and determine if the call may have arisen due to mapping errors, and apply the option provided by LUSTR to process only the non-homologous side when necessary. LUSTR also takes steps to minimize the potential issues arising from mapping by giving flexibility to adjust alignment approaches. For example, in our analyses we noticed that bwa mem automatically adjusts the mapping depth and generated more off-target hits when the reads were mapped to a small target STR list compared to a whole genome scan [69]. One strategy that LUSTR allows is to use a larger STR list for bwa mem, and then focus on the small list for subsequent local realignment and calling. Also, the de novo mapping design of the LUSTR pipeline provides the flexibility for users to easily apply alternative approaches. Alternative mapping methods such as bwa aln or bowtie may work better in the situation where target STRs have homologous loci [69–71] and this can be easily incorporated into LUSTR calling pipeline. Furthermore, LUSTR splits the local realignment and variant calling steps apart and provides intermediate output in plain text format. This design allows for an intermediate checkpoint for users to track the performance and allows for modifications to be made with ease if desired.

LUSTR exhibited great potential in terms of both simulated and real data sets. In addition to LUSTR calling concordant genotypes for > 85% of GIAB benchmark calls, LUSTR also successfully identified several STR variants that were not identified by GIAB published variation calls (VCFs). The variants called by LUSTR were further supported by examining Mendelian inheritance rules, visual inspection of the raw sequence reads (Supplementary Fig. 5), and independent calls by ExpansionHunter (Tables 2 and 3). While the exact reason for the overlooked call is unclear, the difference between LUSTR and GIAB calls may highlight the importance of applying STR specific variant calling tools instead of modified INDEL calling methods as was used for GIAB calling [62]. In evaluating subject samples with expected pathogenic STR variant loci, LUSTR proves its ability and power to apply a whole genome scan to identify disease-causing STR expansions. Using the parallel processing option available in LUSTR, the realignment for the whole genome 168k STRs can be done within days, and the calling step based on the realignment results can be finished within minutes. Among all of the 168k STRs called genome-wide in subject 1, LUSTR successfully identified the expected target, an expansion in the GLS gene, among a small list of high-quality candidate calls. Given that such a result was obtained by an investigation of only three individuals to filter non-specific STR variants, it supports the utility of LUSTR to identify clinically meaningful variants when only a small cohort is available. Furthermore, the performance that LUSTR showed in both detection sensitivity and noise exclusion in just three samples suggests that LUSTR will offer a powerful tool to facilitate large-scale association studies looking for STRs that are associated with disease risk [11]. Besides the GLS STR expansion recognized with top priority, LUSTR also detected several other candidate loci that may contribute to the individual's phenotype. This feature renders it possible to use LUSTR identify oligo- or polygenic risk factors associated with disease [72–75].

While the term “de novo STR mutation” usually indicates the situation when the progeny carries a new STR mutation or pathogenic size expansion not inherited from the parents, the term “novel STR mutation” can be confusing and is often used either in clinical diagnosis or in annotation to distinguish from the term “known STR mutation” [44, 76]. The clinically “novel” STR expansions can refer to newly identified causative expansions without a previous clinical report. The reference-related “novel” STRs, however, indicates repeats that are not present in reference but appear in individuals. Such reference “novel” STRs are challenging to detect by traditional pipelines especially when no preliminary knowledge is available. With more and more attention attracted, several recent tools have been developed to be compatible with “novel” STRs [47, 76, 77]. Aiming to more informative genotyping of each given STR locus, LUSTR focuses on known STRs with repeats available in the reference, with the module for novel STRs to be added in the future updates. Alternatively, when the information is available from clinical reports or from other STR variant calling tools, users can easily customize the list for novel STRs of interest. This limitation of LUSTR explains why it missed the *RFC1* calls in the UDN subjects but was able to detect it later with simple running modifications. The *RFC1* expansion in the two UDN subjects is inherited from an expansion and replacement activity localized to an Alu element, after a nucleotide switch from “AGA” to “GGG” as well as a single nucleotide shift, rendering the reference repetitive unit changed from “AAAAG” to “AAGGG” [78, 79]. It was equivalent to a novel STR expansion and hereby escaped the detection of LUSTR when the reference “AAAAG” repetitive unit was expected, with extremely low numbers of reads able to be realigned. Such coverage warning can serve for users to notice the potential existence of this type of STR mutation and can be scheduled for future updates of LUSTR. However, by simply applying a customized “AAGGG” *RFC1* STR reference or modifying the running with a lower mismatch penalty to allow the realignment of “AAGGG” to “AAAAG”, LUSTR was able to detect the expansion. Furthermore, with such modifications LUSTR identified the inheritance of an “AAAAG” expansion allele and the carriers of heterozygous “AAGGG” expansion allele in the families of two UDN subjects (Supplementary Fig. 6), allowing for further investigation into the potential unrevealed contributions to the phenotype, which so far are suggested to be benign [78–80]. This case indicated the flexibility of LUSTR when applied to complex situations encountered with novel STRs.

## Conclusions

In summary, LUSTR is a reliable and powerful tool for both germline and somatic STR variant calling, and we expect its application to contribute to studies evaluating the role of STR mutations in disease.

## Software availability and requirements

Project name: LUSTR

Project home page: https://github.com/JLuGithub/LUSTR

Operating system: Linux

Programming language: Perl

Other requirements: *samtools*, mapping software such as *bwa* or *bowtie*

License: GNU GPL

Any restrictions to use by non-academics: licence needed

## Method

### LUSTR script

The code for each of the  LUSTR modules were written in Perl script. Regular Smith-Waterman algorithm was applied to the local realignment of short sequences to STR flanking regions. Periodic Smith-Waterman algorithm with modifications was applied to the recognition of STR repeat sequences. The sizes of STR repeats and allele fractions were estimated by calculating the ratios between the counts of reads with and without flanking sequences. The core concept equations are listed below, with modifications applied in practice to allow for random sequencing bias. Equations 1 and 2 are first applied to judge the existence of the allele with repeat length longer than the sequencing read length. Upon the detection of a signal, Equation 3 is used to estimate the size of the repeat region for the allele. The fraction of each allele is then determined by the combination of Equations 4, 5, 6 and 7. The calling reliability was determined by the counts of reads categorized into different patterns and flanking-repeat length distributions under the parameters provided by users. Future updates of LUSTR script will include applications of probability methods to the repeat size estimation, statistics methods to the reliability determination, and functions to incorporate de novo STR variants and long read sequencing libraries.

1 $$\begin{array}{cc}E_{n+1}=1&\lbrack if{(O}_{n+1}>O_n)\&(O_n\geq\sum_{i=1}^n\frac{2S_iC_i}{L-S_i})\rbrack\end{array}$$

2 $$\begin{array}{cc}E_{n+1}=0&(if\ else)\end{array}$$

3 $$\frac{2L{R}_{1}}{{O}_{n+1}-\sum_{i=1}^{n}\frac{2{S}_{i}{C}_{i}}{L-{S}_{i}}}+L\le {S}_{n+1}\le \frac{2L{(R}_{1}+{R}_{2})}{{O}_{n+1}-\sum_{i=1}^{n}\frac{2{S}_{i}{C}_{i}}{L-{S}_{i}}}+L (if\ {E}_{n+1}=1)$$

4 $$\sum\nolimits_{i=1}^{n+1}{F}_{i}=1$$

5 $$\begin{array}{cc}\frac{F_i}{F_j}=\frac{C_i(L-S_j)}{C_j(L-S_i)}&(1\leq i,j\leq n)\end{array}$$

6 $$\begin{array}{cc}\frac{F_{n+1}}{F_i}=\left(O_{n+1}-\sum_{i=1}^n\frac{2S_iC_i}{L-S_i}\right)\cdot\frac{L-S_i}{2C_iL}&(1\leq i\leq n,\ if\ E_{n+1}=1)\end{array}$$

7 $$\begin{array}{cc}\frac{F_{n+1}}{F_i}=0&(1\leq i\leq n,\ if\ E_{n+1}=0)\end{array}$$

L indicates the sequencing read length (bp).

n indicates the number of alleles with repeat sizes (bp) that can be directly detected by reads containing sequences from both flanking regions.

E_n+1_ indicates the existence of the allele (allele n + 1) with repeat size longer than the sequencing read length.

S_i_ (1 ≤ i ≤ n) indicates the repeat size of allele i directly detected by reads; S_n+1_ indicates the repeat size of allele n + 1 that is longer than the sequencing read length thus needs to be estimated.

C_i_ (1 ≤ i ≤ n) indicates the number of reads containing sequences from both flanking regions and a repeat region with size of S_i_, thus belonging to allele i.

O_n_ indicates the number of reads containing sequences from only one flanking region, and from the pairs with any repeat length ≤ the maximum from S_1_ to S_n_; O_n+1_ indicates the number of all of the reads containing sequences from only one flanking region.

F_i_ (1 ≤ i ≤ n) indicates the fraction of allele i; F_n+1_ indicates the fraction of allele n + 1 whose repeat size is longer than the sequencing read length.

R_1_ indicates the number of reads containing only repeat sequences but not from repeat-only pairs; R_2_ indicates the number of reads containing only repeat sequences and from repeat-only pairs.

### Data processing

The running of LUSTR and the processing of the short read sequencing libraries were done in Linux, with SAMTOOLS 1.14 pre-installed. The mapping of reads to STR references was done by BWA MEM version 0.7.

### Data generation

The simulated data in the performance test of LUSTR was generated by in-house Perl script. STR references with expected repeat sizes were prepared, and then read pairs were generated in random directions from the STR references. The pattern of each read was recorded to evaluate the performance of LUSTR calling. Each nucleotide of reads was by a given chance altered, deleted, or inserted to imitate sequencing errors.

The mixed library data was generated by an in-house Perl script. 

### Supplementary Information

**Additional file 1: Supplementary Figure 1.** Structure of C9orf72 STR. We show here the reference sequence surrounding an STR within *C9orf72* as a typical example of the complexities of STR structure. This STR has been reported to be associated with amyotrophic lateral sclerosis (ALS) and contains GGCCCC repeats. It is located on chromosome 9, and the genomic location (build 37) is shown in the figure. The approximate boundaries between the repeat and flanking regions are indicated. This figure shows how allowing incomplete repeats and tolerating repeat mismatches can greatly influence how one defines the repeat region that will be interrogated in the downstream models to infer genotype. *Note that the algorithm is agnostic to strand. For this *C9orf72* STR, inputting CCGGGG from the reverse strand will be treated as equivalent to indicating CCCCGG from the forward strand.

**Additional file 2: Supplementary Figure 2.** Determination of the repeat sequence of C9orf72 STR by LUSTR applying periodic Smith-Waterman algorithm. We show here as an example how the LUSTR finder module determines the repeat sequence of *C9orf72* STR by applying the periodic Smith-Waterman algorithm, searching for GGCCCC repetitive sequences using the default settings as follows: match/mismatch/gap/stop = 2/-5/-7/-30. Starting from the seed sequence (two GGCCCC repeats, highlighted in yellow), the finder module aligns the reference periodically to GGCCCC in both upstream and downstream directions and records the best score at each nucleotide. Scores above 0 will be reset to 0, and routines with a score below the stop limit will be blocked for further extension. In this case, the extension stops when the best score is below -30 (highlighted in orange), and the repeat sequence is determined by the farthest nucleotides with a score of 0 (highlighted in green).

**Additional file 3: Supplementary Figure 3.** Average read coverage of 13 STR loci in GIAB trios. Average read coverage by GIAB trio libraries for the 13 STR loci tested in this study. Reads from each individual or merged library were first mapped to the whole human genome by bwa mem. Coverage of each nucleotide within the STR loci region (repeat region plus 2 x 50 bp flanking sequence at both sides) was calculated by SAMTOOLS depth, and the average coverage of each STR locus was calculated. STRs with failed or allele-missing calls in certain libraries are indicated by red color.

**Additional file 4: Supplementary Figure 4.** Reads realigned to ATN1 and HTT STR loci from the son of GIAB Ashkenazim trio. Raw sequences of the reads realigned to the two loci were collected from the libraries sequenced for the Ashkenazim son. Gaps are indicated, and mismatched nucleotides are marked in red. Reads are categorized according to their repeat sizes. Interestingly, besides the dominant alleles, LUSTR identified one read directly supporting the -5 allele at ATN1 STR locus, and one read directly supporting the -9 allele at HTT STR locus. These reads might indicate potential small fraction somatic STR variants, but further confirmation is needed to exclude the possibility of random sequencing error.

**Additional file 5: Supplementary Figure 5.** Reads supporting the STR alleles called by LUSTR but not revealed in GIAB database. Raw sequences of the reads realigned to (a) ATXN3 STR locus in father and son from the Ashkenazim trio, (b) DMPK STR locus in mother and son from the Ashkenazim trio, (c) DMPK STR locus in mother from the Chinese trio, and (d) PPP2R2B STR locus in father and mother from the Chinese trio. Gaps are indicated, and mismatched nucleotides are marked in red. Reads are categorized according to their repeat sizes.

**Additional file 6: Supplementary Figure 6.** Potential inheritance of RFC1 STR alleles in the families of UDN subject 1 and 2. The genotypes of RFC1 STR alleles identified by LUSTR are shown for the pedigrees of UDN families of subject 1 and subject 2, for whom nuclear family members were available. The reference RFC1 STR allele (AAAAG wt, marked in blue) has two mutant types, AAAAG expansion (marked in orange and not known to be associated with disease) and AAGGG expansion (marked in red). The alleles were confirmed by checking the raw reads in sequenced libraries.

**Additional file 7: Supplementary Table 1.** a Performance of LUSTR in identification of STR variants in GIAB database (Ashkenazim Trio). b Performance of LUSTR in identification of STR variants in GIAB database (Chinese Trio). **Supplementary Table 2.** a Evaluation of candidate STR expansions by LUSTR unbiased whole genome scan for subject 2. b Evaluation of candidate STR expansions by LUSTR unbiased whole genome scan for subject 3. **Supplementary Table 3.** a RFC1 expansion calls by LUSTR with alternative references for subject 2. b RFC1 expansion calls by LUSTR with alternative references for subject 3. **Supplementary Table 4.** Comparison among LUSTR, ExpansionHunter, and GangSTR

**Additional file 8.** Full list of Undiagnosed Disease Network members.

**Additional file 9.** A .zip file including following LUSTR scripts, which can also be downloaded from https://github.com/JLuGithub/LUSTR: LUSTR_Finder.pl, LUSTR_RefCreator.pl, LUSTR_Extractor.pl, LUSTR_Realigner.pl, LUSTR_Caller.pl, README.txt, README.md, README_detail.txt, QuickGuide.txt, LICENSE.txt, testdata/test_genome_hg19_chr9_27571483_27575544.fa, testdata/test_pairendreads_C9orf72_ref70exp30.fastq, testdata/test_STRinfo.txt.

## Data Availability

The raw read libraries and variant calling result files from the GIAB project were downloaded from ftp://ftp-trace.ncbi.nih.gov/giab/ftp/. The raw read libraries UDN project were obtained directly from NIH Undiagnosed Diseases Program by collaboration.

## Abbreviations

STRs: Short tandem repeats
INDELs: Insertions or deletions
ALS: Amyotrophic lateral sclerosis
PCR: Polymerase chain reaction
GIAB: Genome in a Bottle Consortium
VCFs: Variant calling files
UDN: Undiagnosed Disease Network
T2T: Telomere-to-Telomere genome reference
