# Supplementary figures and images for: LUSTR: a new customizable tool for calling genome-wide germline and somatic short tandem repeat variants
Source: BMC Genomics. 2024 Jan 26;25:115. doi: 10.1186/s12864-023-09935-9 (PMC10811831; doi:10.1186/s12864-023-09935-9)

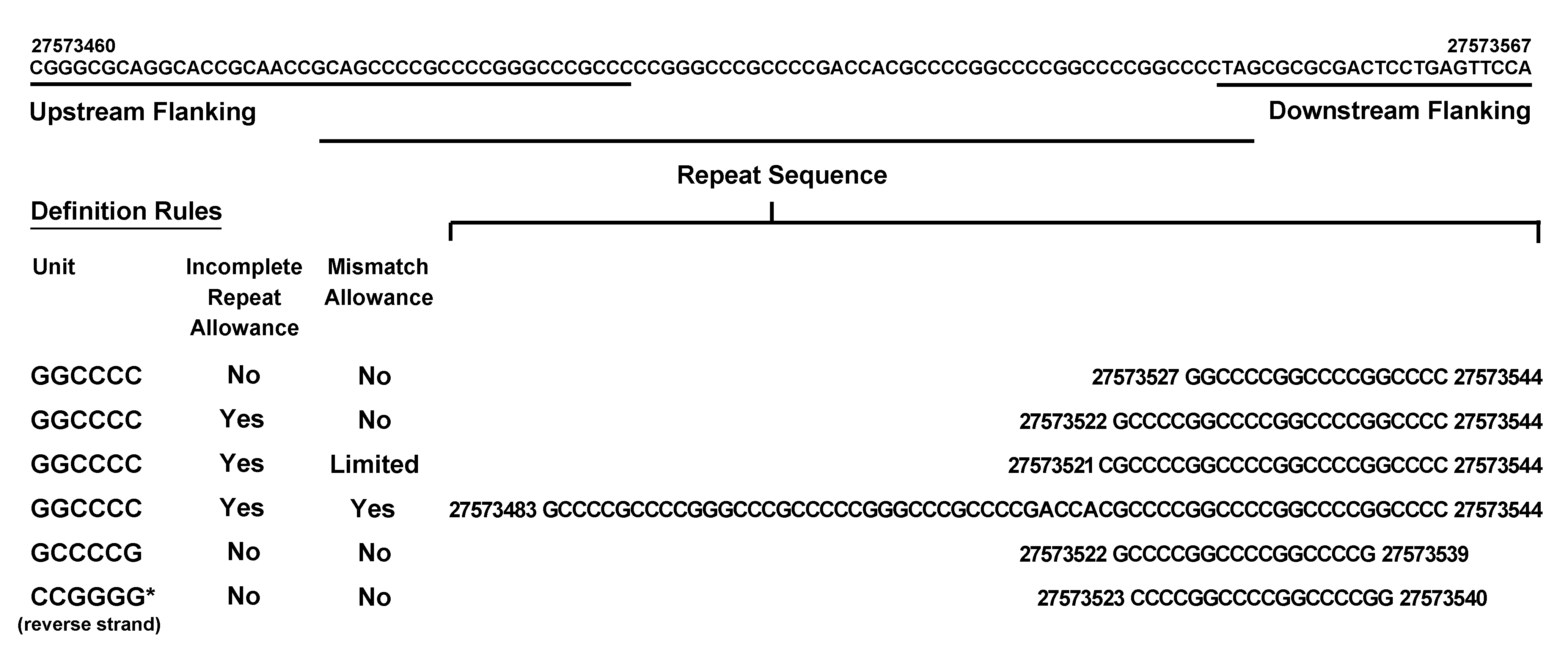

Supplement: Supplementary file 1 — Additional file 1: Supplementary Figure 1. Structure of C9orf72 STR. We show here the reference sequence surrounding an STR within C9orf72 as a typical example of the complexities of STR structure. This STR has been reported to be associated with amyotrophic lateral sclerosis (ALS) and contains GGCCCC repeats. It is located on chromosome 9, and the genomic location (build 37) is shown in the figure. The approximate boundaries between the repeat and flanking regions are indicated. This figure shows how allowing incomplete repeats and tolerating repeat mismatches can greatly influence how one defines the repeat region that will be interrogated in the downstream models to infer genotype. *Note that the algorithm is agnostic to strand. For this C9orf72 STR, inputting CCGGGG from the reverse strand will be treated as equivalent to indicating CCCCGG from the forward strand. [file 12864_2023_9935_MOESM1_ESM.tif]

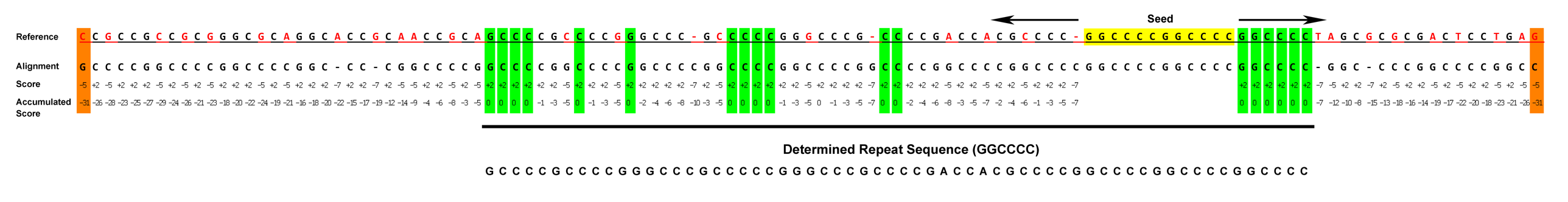

Supplement: Supplementary file 2 — Additional file 2: Supplementary Figure 2. Determination of the repeat sequence of C9orf72 STR by LUSTR applying periodic Smith-Waterman algorithm. We show here as an example how the LUSTR finder module determines the repeat sequence of C9orf72 STR by applying the periodic Smith-Waterman algorithm, searching for GGCCCC repetitive sequences using the default settings as follows: match/mismatch/gap/stop = 2/-5/-7/-30. Starting from the seed sequence (two GGCCCC repeats, highlighted in yellow), the finder module aligns the reference periodically to GGCCCC in both upstream and downstream directions and records the best score at each nucleotide. Scores above 0 will be reset to 0, and routines with a score below the stop limit will be blocked for further extension. In this case, the extension stops when the best score is below -30 (highlighted in orange), and the repeat sequence is determined by the farthest nucleotides with a score of 0 (highlighted in green). [file 12864_2023_9935_MOESM2_ESM.tif]

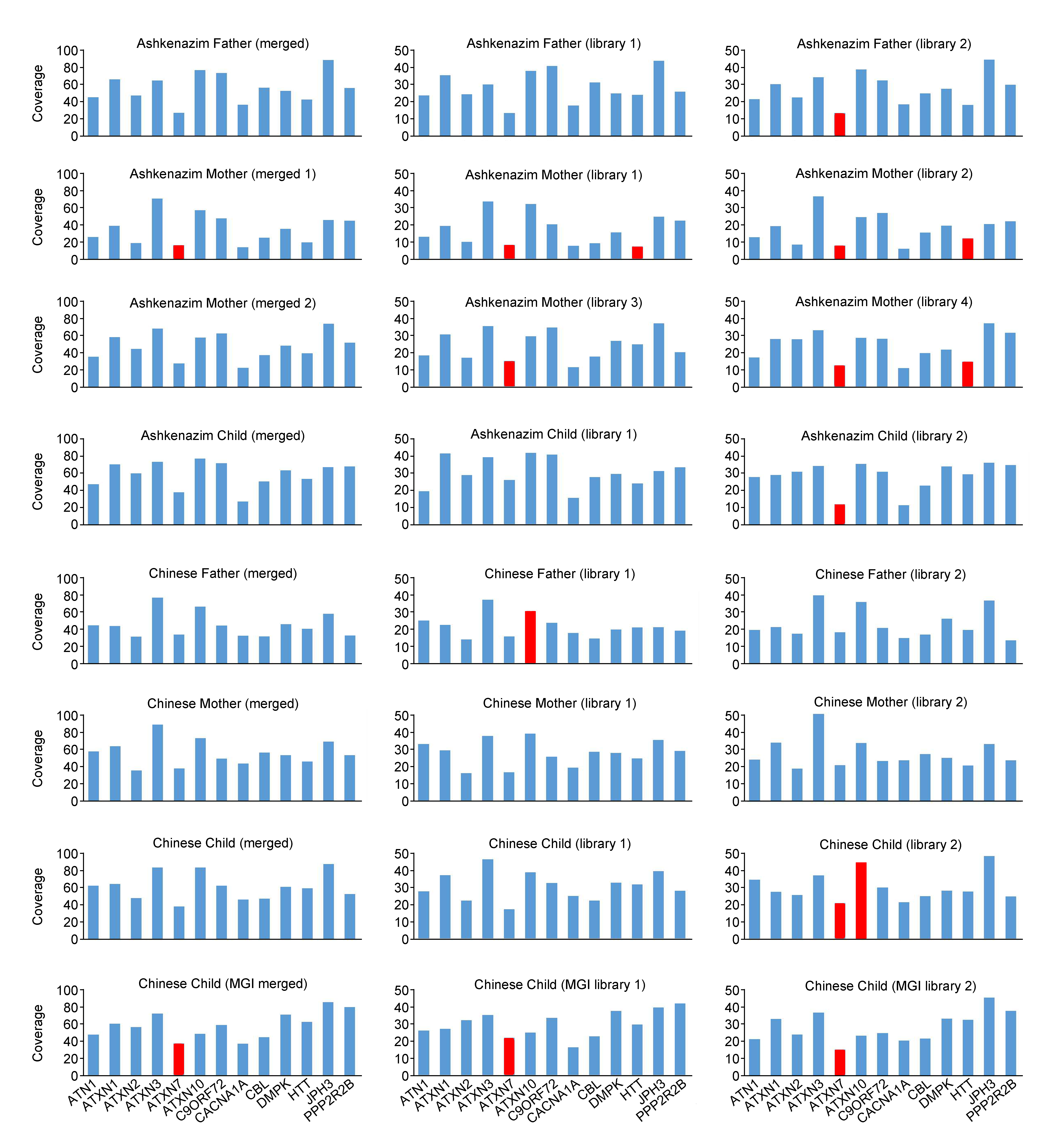

Supplement: Supplementary file 3 — Additional file 3: Supplementary Figure 3. Average read coverage of 13 STR loci in GIAB trios. Average read coverage by GIAB trio libraries for the 13 STR loci tested in this study. Reads from each individual or merged library were first mapped to the whole human genome by bwa mem. Coverage of each nucleotide within the STR loci region (repeat region plus 2 x 50 bp flanking sequence at both sides) was calculated by SAMTOOLS depth, and the average coverage of each STR locus was calculated. STRs with failed or allele-missing calls in certain libraries are indicated by red color. [file 12864_2023_9935_MOESM3_ESM.tif]

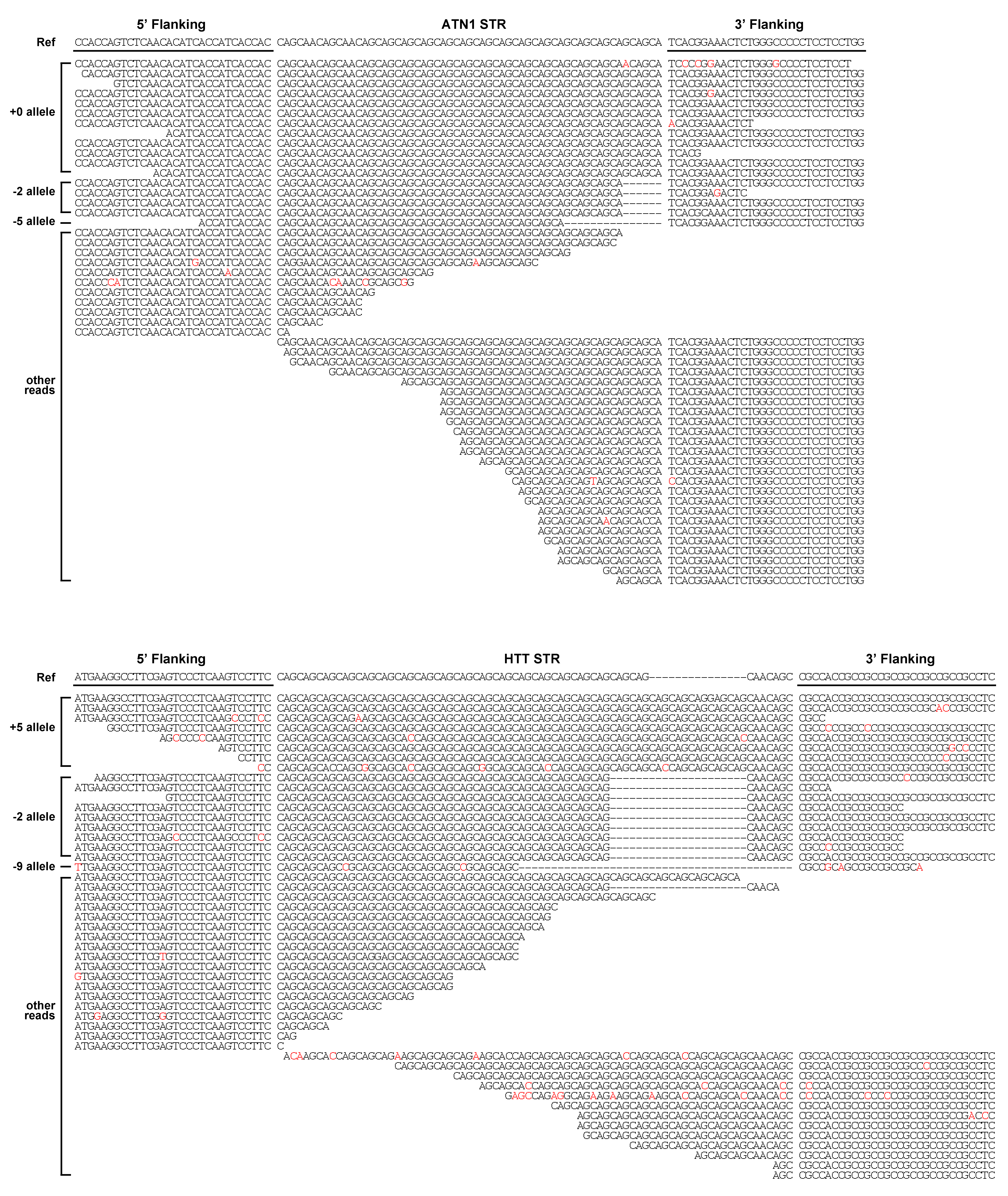

Supplement: Supplementary file 4 — Additional file 4: Supplementary Figure 4. Reads realigned to ATN1 and HTT STR loci from the son of GIAB Ashkenazim trio. Raw sequences of the reads realigned to the two loci were collected from the libraries sequenced for the Ashkenazim son. Gaps are indicated, and mismatched nucleotides are marked in red. Reads are categorized according to their repeat sizes. Interestingly, besides the dominant alleles, LUSTR identified one read directly supporting the -5 allele at ATN1 STR locus, and one read directly supporting the -9 allele at HTT STR locus. These reads might indicate potential small fraction somatic STR variants, but further confirmation is needed to exclude the possibility of random sequencing error. [file 12864_2023_9935_MOESM4_ESM.tif]

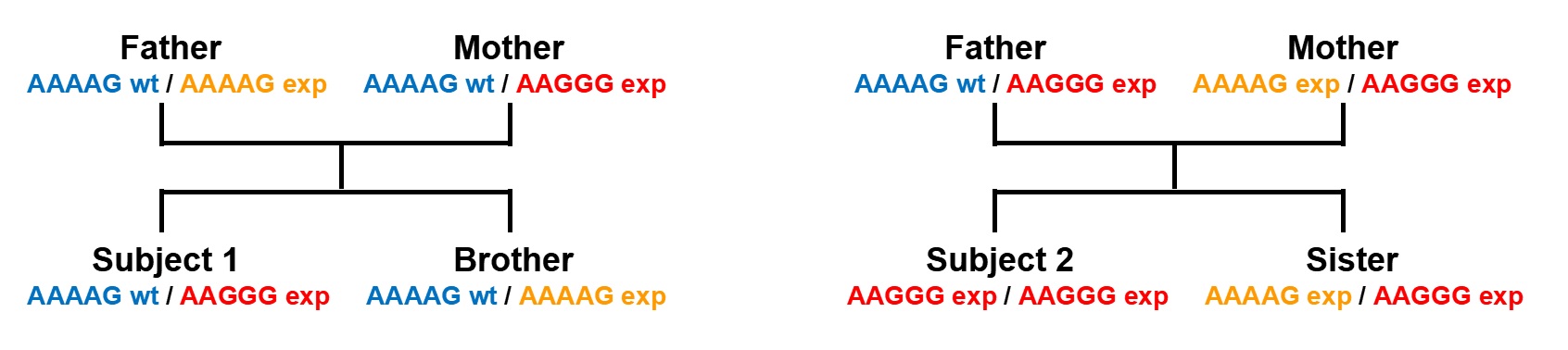

Supplement: Supplementary file 6 — Additional file 6: Supplementary Figure 6. Potential inheritance of RFC1 STR alleles in the families of UDN subject 1 and 2. The genotypes of RFC1 STR alleles identified by LUSTR are shown for the pedigrees of UDN families of subject 1 and subject 2, for whom nuclear family members were available. The reference RFC1 STR allele (AAAAG wt, marked in blue) has two mutant types, AAAAG expansion (marked in orange and not known to be associated with disease) and AAGGG expansion (marked in red). The alleles were confirmed by checking the raw reads in sequenced libraries. [file 12864_2023_9935_MOESM6_ESM.jpg]
