# Supplementary material for: LUSTR: a new customizable tool for calling genome-wide germline and somatic short tandem repeat variants
Source: BMC Genomics. 2024 Jan 26;25:115. doi: 10.1186/s12864-023-09935-9 (PMC10811831; doi:10.1186/s12864-023-09935-9)
Supplement: Supplementary file 7 — Additional file 7: Supplementary Table 1. a Performance of LUSTR in identification of STR variants in GIAB database (Ashkenazim Trio). b Performance of LUSTR in identification of STR variants in GIAB database (Chinese Trio). Supplementary Table 2. a Evaluation of candidate STR expansions by LUSTR unbiased whole genome scan for subject 2. b Evaluation of candidate STR expansions by LUSTR unbiased whole genome scan for subject 3. Supplementary Table 3. a RFC1 expansion calls by LUSTR with alternative references for subject 2. b RFC1 expansion calls by LUSTR with alternative references for subject 3. Supplementary Table 4. Comparison among LUSTR, ExpansionHunter, and GangSTR [file 12864_2023_9935_MOESM7_ESM.doc]

**Supplementary Table 1a** **Performance of LUSTR in** **identification of STR variations in GIAB database (Ashkenazim Trio)**

| Loci | Father | | | Mother | | | | | Child | | |
| --- | --- | --- | --- | --- | --- | --- | --- | --- | --- | --- | --- |
|  | Call by  GIAB | Call by LUSTR  (library 1) | Call by LUSTR  (library 2) | Call by  GIAB | Call by LUSTR  (library 1) | Call by LUSTR  (library 2) | Call by LUSTR  (library 3) | Call by LUSTR  (library 4) | Call by  GIAB | Call by LUSTR  (library 1) | Call by LUSTR  (library 2) |
|  |  |  |  |  |  |  |  |  |  |  |  |
| ATN1 (CAG)  12:7045880-938 | NA | 0 (100±0%) | 0 (100±0%) | -7/-2 | -7 (57±57%)  -2 (43±57%) | -7 (30±48%)  -2 (35±47%)  -1.7 (35±47%) | -7 (34±38%)  -2 (66±38%) | -7 (30±51%)  -2 (70±51%) | -2/0 | -5 (13±40%)  0 (87±40%) | -2 (38±31%)  0 (62±31%) |
| ATXN1 (TGC)  6:16327865-955 | 0/+1 | 0 (83±51%)  +1 (17±51%) | 0 (69±31%)  +1 (31±31%) | 0/+1 | -1 (18±60%)  +1 (82±60%) | -1 (47±39%)  +1 (53±39%) | -1 (31±51%)  +1 (69±51%) | -1 (27±42%)  +1 (73±42%) | 0/+1 | 0 (46±16%)  +1 (54±16%) | 0 (21±33%)  +1 (79±33%) |
| ATXN2 (GCT)  12:112036754-823 | -1/+7 | -1 (74±59%)  +7 (26±59%) | -1 (12±47%)  +7 (88±47%) | NA | -1 (100±0%) | -1 (100±0%) | -1 (100±0%) | -1 (88±41%) | NA | -1 (49±51%)  +7 (51±51%) | -1 (64±42%)  +7 (36±42%) |
| ATXN3 (CTG)  14:92537355-96 | NA | +6.7 (51±16%)  +9 (49±16%) | +6.7 (65±21%)  +9 (35±21%) | 0/+13 | 0 (51±27%)  +12.7 (20±29%)  +13 (30±30%) | 0 (56±20%)  +13 (44±20%) | 0 (56±17%)  +13 (44±17%) | 0 (64±21%)  +12.7 (36±21%) | NA | +9 (65±17%)  +13 (35±17%) | +9 (56±22%)  +12.7 (44±22%) |
| ATXN7 (GCA)  3:63898361-423 | NA | 0 (100±0%) | NA | 0/+3 | NA | +3 (100±0%) | +2.7 (100±0%) | 0 (100±0%) | 0/+3 | 0 (31±69%)  +3 (69±69%) | +3 (100±0%) |
| ATXN10 (ATTCT)  22:46191235-304 | 0/+2 | 0 (50±18%)  +2 (50±18%) | 0 (74±15%)  +2 (26±15%) | -2/-1 | -2 (49±15%)  -1 (51±15%) | -2 (40±25%)  -1 (60±25%) | -2 (62±16%)  -1 (38±16%) | -2 (68±19%)  -1 (32±19%) | -2/+2 | -2 (62±12%)  +2 (38±12%) | -2 (55±14%)  +2 (45±14%) |
| C9ORF72 (GCCCCG)  9:27573483-544 | -1/-1 | -1 (100±0%) | -1 (100±0%) | -1/-1 | -1 (100±0%) | -1 (100±0%) | -1 (100±0%) | -1 (100±0%) | -1/-1 | -1 (100±0%) | -1 (100±0%) |
| CACNA1A (CTG)  19:13318673-712 | -2/-1 | -2 (66±66%)  -1 (34±66%) | -2 (83±50%)  -1 (17±50%) | NA | -2 (100±0%) | NA | -1 (100±0%) | NA | -2/-2 | -2 (100±0%) | -2 (100±0%) |
| CBL (CGG)  11:119077000-33 | 0/+5 | 0 (50±21%)  +5 (50±21%) | 0 (26±43%)  +5 (74±43%) | NA | 0 (100±0%) | 0 (56±56%) | 0 (86±42%) | 0 (100±0%) | NA | 0 (100±0%) | 0 (40±40%) |
| DMPK (CAG)  19:46273463-524 | -9/-7 | -9 (46±16%)  -7 (54±16%) | -9 (56±12%)  -7 (44±12%) | NA | -15 (74±19%)  -9 (26±19%) | -15 (54±17%)  -9 (46±17%) | -15 (46±12%)  -9 (54±12%) | -15 (44±16%)  -9 (56±16%) | NA | -9 (100±0%) | -9 (100±0%) |
| HTT (CAG)  4:3076604-67 | -2/-2 | -2 (100±0%) | -2 (100±0%) | -2/+5 | +5 (100±0%) | -2 (100±0%) | -2 (61±48%)  +5 (39±48%) | -2 (100±0%) | -2/+5 | -9 (7±25%)  -2 (26±30%)  +5 (67±31%) | -2 (70±38%)  +5 (30±38%) |
| JPH3 (GCT)  16:87637889-935 | 0/+2 | 0 (56±11%)  +2 (44±11%) | 0 (55±13%)  +2 (45±13%) | 0/+2 | 0 (79±21%)  +2 (21±21%) | 0 (79±21%)  +2 (21±21%) | 0 (59±15%)  +2 (41±15%) | 0 (49±13%)  +2 (52±13%) | +2/+2 | +2 (100±0%) | +2 (100±0%) |
| PPP2R2B (GCT)  5:146258291-322 | NA | 0 (100±0%) | 0 (100±0%) | NA | 0 (100±0%) | 0 (100±0%) | 0 (100±0%) | 0 (100±0%) | NA | 0 (100±0%) | 0 (100±0%) |

**Supplementary Table 1b**  **Performance of LUSTR in** **identification of STR variations in GIAB database (Chinese Trio)**

| Loci | Father | | | Mother | | | Child | | | | |
| --- | --- | --- | --- | --- | --- | --- | --- | --- | --- | --- | --- |
|  | Call by  GIAB | Call by LUSTR  (library 1) | Call by LUSTR  (library 2) | Call by  GIAB | Call by LUSTR  (library 1) | Call by LUSTR  (library 2) | Call by  GIAB | Call by LUSTR  (library 1) | Call by LUSTR  (library 2) | Call by LUSTR  (MGI library 1) | Call by LUSTR  (MGI library 2) |
|  |  |  |  |  |  |  |  |  |  |  |  |
| ATN1 (CAG)  12:7045880-938 | -5/+4 | -5 (51±49%)  +4 (49±49%) | -5 (17±39%)  +4 (83±39%) | NA | 0 (49±18%)  +2 (51±18%) | 0 (46±40%)  +2 (54±40%) | NA | 0 (35±46%)  +4 (65±46%) | 0 (19±35%)  +4 (81±35%) | 0 (59±41%)  +4 (41±41%) | 0 (33±27%)  +4 (67±27%) |
| ATXN1 (TGC)  6:16327865-955 | NA | -3 (40±60%)  -1 (60±60%) | -3 (40±60%)  -1 (60±60%) | NA | -1 (33±36%)  [+5,+21] (67±36%) | +3 (84±16%) | NA | -1 (100±0%) | -1 (100±0%) | -1 (100±0%) | -1 (100±0%) |
| ATXN2 (GCT)  12:112036754-823 | NA | -4 (16±60%)  -1 (84±60%) | -1 (100±0%) | NA | -1 (100±0%) | -1 (22±35%) | NA | -1 (100±0%) | -1 (100±0%) | -1 (73±73%) | -1 (67±67%) |
| ATXN3 (CTG)  14:92537355-96 | NA | 0 (100±0%) | 0 (100±0%) | 0/+6 | 0 (82±24%)  +6 (18±24%) | 0 (51±9%)  +6 (49±9%) | NA | 0 (100±0%) | 0 (100±0%) | 0 (100±0%) | 0 (100±0%) |
| ATXN7 (GCA)  3:63898361-423 | NA | 0 (100±0%) | 0 (100±0%) | NA | 0 (63±49%)  +2 (37±49%) | 0 (30±70%)  +2 (70±70%) | 0/+2 | 0 (72±72%)  +2 (28±72%) | 0 (100±0%) | 0 (100±0%) | NA |
| ATXN10 (ATTCT)  22:46191235-304 | +3/+7 | +3 (100±0%) | +2.8 (35±38)  +3 (38±38%)  +10.1 (27±20%) | NA | -0.2 (38±23%)  0 (62±23%) | 0 (100±0%) | NA | 0 (43±57%)  +4.2 (57±57%) | -0.2 (35±21%)  0 (65±21%) | 0 (48±52%)  +7 (52±52%) | 0 (12±57%)  +7 (88±57%) |
| C9ORF72 (GCCCCG)  9:27573483-544 | NA | -1 (100±0%) | -1 (100±0%) | -1/+3 | -1 (37±61%)  +3 (61±61%)  +40 (2±16%) | -1 (43±57%)  +3 (57±57%) | -1/-1 | -1 (100±0%) | -1 (51±19%) | -1 (100±0%) | -1 (100±0%) |
| CACNA1A (CTG)  19:13318673-712 | NA | 0 (40±26%)  +1 (60±26%) | 0 (74±74%)  +1 (26±74%) | NA | 0 (100±0%) | 0 (100±0%) | NA | 0 (100±0%) | 0 (100±0%) | 0 (100±0%) | NA |
| CBL (CGG)  11:119077000-33 | 0/+1 | 0 (43±34%)  +1 (57±34%) | 0 (90±31%)  +1 (10±31%) | NA | 0 (100±0%) | 0 (100±0%) | NA | 0 (100±0%) | 0 (100±0%) | 0 (100±0%) | 0 (100±0%) |
| DMPK (CAG)  19:46273463-524 | -5/-4 | -5 (49±38%)  -4 (51±38%) | -5 (60±24%)  -4 (40±24%) | NA | -7 (100±0%) | -7 (100±0%) | -7/-4 | -7 (32±17%)  -4 (68±17%) | -7 (55±18%)  -4 (45±18%) | -7 (54±14%)  -4 (46±14%) | -7 (50±17%)  -4 (50±17%) |
| HTT (CAG)  4:3076604-67 | -2/-1 | -2 (13±42%)  -1 (87±42%) | -2 (24±76%)  -1 (76±76%) | -2/-1 | -2 (32±50%)  -1 (68±50%) | -2 (27±43%)  -1 (73±43%) | -1/-1 | -1 (92±22%)  0 (8±22%) | -1 (100±0%) | -1 (100±0%) | -1 (100±0%) |
| JPH3 (GCT)  16:87637889-935 | -1/+2 | -1 (33±39%)  +2 (67±39%) | -1 (46±19%)  +2 (54±19%) | NA | 0 (100±0%) | 0 (100±0%) | 0/+2 | 0 (24±20%)  +2 (76±20%) | 0 (33±17%)  +2 (67±17%) | 0 (31±12%)  +2 (69±12%) | 0 (42±10%)  +2 (58±10%) |
| PPP2R2B (GCT)  5:146258291-322 | NA | +6 (47±22%)  +8 (53±22%) | +6 (67±33%)  +8 (33±33%) | NA | +3 (66±34%)  +6 (34±34%) | +3 (30±24%)  +6 (70±24%) | +3/+6 | +3 (68±16%)  +6 (32±16%) | +3 (46±40%)  +6 (54±40%) | +3 (48±14%)  +6 (52±14%) | +3 (41±14%)  +6 (59±14%) |

NA = not available, genotype not provided by GIAB in the VCF or not able to be called by LUSTR. Note unlike LUSTR, we could not distinguish between reference genotype called or no call provided from GIAB data. To compare, we assume that the NAs from GIAB data are reference genotypes and consistent with LUSTR results if supported by LUSTR calls, while the NAs from GIAB data are discrepant from LUSTR results if the LUSTR calls are distant from reference genotypes. Name, repeat unit, and location in genome (build 37) of each STR are shown in the first column. STR variations expected by GIAB calls or genotyped by LUSTR are shown as repeat number changes compared to reference (build 37). Alleles called by LUSTR are shown by each line, and expansions called by estimation are shown as a range in square brackets. The estimated allele fraction and uncertainty range by LUSTR are shown in brackets following each correlated genotyped allele. Two or four independent <double check> libraries for each member in the two trios were downloaded and analyzed separately for this test. All libraries of the Ashkenazim trio were sequenced by MGISEQ platform. All libraries of the Chinese trio were sequenced by BGISEQ platform except two libraries of the child which were sequenced by MGISEQ.

**Supplementary Table 2a Evaluation of candidate STR expansions by LUSTR unbiased whole genome scan for subject 2**

| STR | Affected | Region | Distance to | Reference | LUSTR Warning^1^ | Specificity^2^ | | Other Information for Evaluation | Priority^3^ |
| --- | --- | --- | --- | --- | --- | --- | --- | --- | --- |
|  | Gene |  | nearest exon | Repeat Size |  | Subject 1 | Subject 3 |  |  |
| 1  2  3  4  5  6  7  8  9  10  11  12  13  14  15  16  17  18  19  20  21 | KLHL23  CCSER1  FAM20C  LOC100506990  FAM49B  ROR2  STK33  MYEOV  ARHGAP42  CADM1  MGAT4C  LINC00639  RAB37  BAHCC1  TCF4  INSR  MIR549XHG  TMPRSS6  GLRA2  CHM  AFF2 | exon  intron  intron  intron  intron  intron  intron  intron  intron  intron  intron  intron  intron  intron  intron  intron  intron  intron  intron  intron  intron | 0bp  far away  100bp  4kb  far away  1kb  4kb  far away  far away  far away  far away  far away  3kb  far away  30bp  500bp  far away  4kb  far away  20bp  far away | long  short  long  short  short  long  long  long  short  short  short  short  long  long  short  long  short  short  short  short  short | -  -  -  -  -  -  -  3’ flanking mutant  5’ flanking mutant  -  -  -  -  -  -  -  -  -  -  -  - | smaller  smaller  smaller  also called  smaller  smaller  smaller  smaller  smaller  also called  also called  -  smaller  also called  -  -  also called  -  also called  also called  - | smaller  smaller  smaller  smaller  smaller  smaller  -  also called  smaller  smaller  smaller  low quality  smaller  also called  -  low %  -  -  low quality  smaller  also called | -  -  -  -  -  -  also called but smaller in Sister  -  -  -  -  also called in Mother, multiallelic calls  -  -  also called in Mother & Sister, 5’ flank repeats  low % call in one repeat  also called in Mother  also called in Sister & smaller calls in parents  also called in all family members  -  also called in all family members | ●  -  ○  ●  -  -  ○  -  -  -  -  ●  -  -  ●●○  ●  ○  ●●  -  ●○  - |

**Supplementary Table 2b Evaluation of candidate STR expansions by LUSTR unbiased whole genome scan for subject 3**

| STR | Affected | Region | Distance to | Reference | LUSTR Warning^1^ | Specificity^2^ | | Other Information for Evaluation | Priority^3^ |
| --- | --- | --- | --- | --- | --- | --- | --- | --- | --- |
|  | Gene |  | nearest exon | Repeat Size |  | Subject 1 | Subject 2 |  |  |
| 1 | ZNF180 | intron | 6kb | long | - | smaller | also called | - | - |

Details of the 21 primary STR expansion candidates for subject 2 (a) and the 1 primary STR expansion candidate for subject 3 (b) in the unbiased whole genome scan by LUSTR (Table 4).

^1^ Indicating the warning messages given by LUSTR for potential homologous flanking sequences or mutations within flanking regions close to the repeats

^2^ Indicating whether the expansion was also called in other subjects. The calls in other subjects did not trigger previous filtration because they were: (1) not called by both other subjects; (2) called by low qualities; or (3) called by low fractions or smaller size variations that didn’t pass the threshold settings

^3^ The priority of each candidate STR expansion was determined based on the information collected in this table. The number of ● and ○ indicates the level of priority, where ○ indicate a lower priority contribution compared to ●. Noting that this priority did not necessarily mean true or false positive, but rather served as a guidance for further evaluation and confirmation based on raw reads inspection, targeted sequencing, and potential clinical importance

**Supplementary Table 3a RFC1 expansion calls by LUSTR with alternative references for subject 2**

| RFC1 | Original (REF = 12.4, AAAAG, mis = -5) | | | Alter 1 (REF = 11.6, AAGGG, mis = -5) | | | Alter 2 (REF = 12.6, AAAAG, mis = -3) | | |
| --- | --- | --- | --- | --- | --- | --- | --- | --- | --- |
| 4:39350044-39350105 | Call | Quality | Pairs realigned | Call | Quality | Pairs realigned | Call | Quality | Pairs realigned |
|  |  |  |  |  |  |  |  |  |  |
| Subject 2 | [+17.8,+78.2] (100±0%) | Low | 21 | +38.7 (100±0%) | High | 52 | [+17.6,+40.5] (100±0%) | Low | 43 |
| Father | 0 (100±0%) | High | 56 | [+34.3,+38.7] (100±0%) | High | 32 | 0 (75±7%)  [+17.6,+109.8] (25±7%) | High  Low | 70 |
| Mother | [+34.3,+122.1] (100±0%) | High | 43 | +66.9 (100±0%) | Medium | 18 | [+34.3,+113.4] (100±0%) | High | 53 |
| Sister | [+41.6,+114.8] (100±0%) | High | 59 | +34.9 (100±0%) | Medium | 27 | [+36.4,+98.1] (100±0%) | High | 73 |

**Supplementary Table 3b RFC1 expansion calls by LUSTR with alternative references for subject 3**

| RFC1 | Original (REF = 12.4, AAAAG, mis = -5) | | | Alter 1 (REF = 11.6, AAGGG, mis = -5) | | | Alter 2 (REF = 12.6, AAAAG, mis = -3) | | |
| --- | --- | --- | --- | --- | --- | --- | --- | --- | --- |
| 4:39350044-39350105 | Call | Quality | Pairs realigned | Call | Quality | Pairs realigned | Call | Quality | Pairs realigned |
|  |  |  |  |  |  |  |  |  |  |
| Subject 3 | [+17.8,+31.2] (100±0%) | Low | 10 | [+32,+37.4] (100±0%) | High | 51 | [+17.6,+23.5] (100±0%) | Medium/Low | 41 |

Libraries of subject 2, 3 and the family members (if available) were rerun by LUSTR with customized RFC1 STR references, by modifying either the repeat sequences (Alter 1) or the score for mismatch (Alter 2). Libraries from the same individual were merged. Reads were first remapped to the full list of STRs to reduce offtarget interference, and were then processed by targeted realignment and calling using LUSTR for RFC1 STR locus. Called repeat number changes compared to reference, allele fraction estimations, qualities, and numbers of pairs realigned for each RFC1 STR reference are shown. Alleles called by LUSTR are shown by each line, and expansions called by estimation are shown as a range in square brackets.

**Supplementary Table 4 Comparison among LUSTR, ExpansionHunter, and GangSTR**

|  | ExpansionHunter | GangSTR | LUSTR |
| --- | --- | --- | --- |
|  |  |  |  |
| Output | VCF | VCF | Plain text |
| Target Customization effort | from Medium (format requirement)  to Hard (offtarget information) | from Easy  to Hard (offtarget information) | Easy |
| Calling Mode | Germline | Germline | Somatic |
| Running Speed (Single thread) | from Fast  to Medium (with offtarget information or due to problematic loci) | from Fast  to Medium (with offtarget information) | Slow |
| Multithreading option | Yes | No | Yes |

A comparison among LUSTR and two other widely used existing STR variation calling tools, ExpansionHunter and GangSTR. The comparison shows differences in input/output requirements, running options and performance. Brief explanations were provided in parentheses.
