# Supplementary material for: LUSTR: a new customizable tool for calling genome-wide germline and somatic short tandem repeat variants
Source: BMC Genomics. 2024 Jan 26;25:115. doi: 10.1186/s12864-023-09935-9 (PMC10811831; doi:10.1186/s12864-023-09935-9)
Supplement: Supplementary file 8 — Additional file 8. Full list of Undiagnosed Disease Network members. [file 12864_2023_9935_MOESM8_ESM.docx]

**Members of the Undiagnosed Diseases Network**

Maria T. Acosta

Margaret Adam

David R. Adams

Raquel L. Alvarez

Justin Alvey

Laura Amendola

Ashley Andrews

Euan A. Ashley

Carlos A. Bacino

Guney Bademci

Ashok Balasubramanyam

Dustin Baldridge

Jim Bale

Michael Bamshad

Deborah Barbouth

Pinar Bayrak-Toydemir

Anita Beck

Alan H. Beggs

Edward Behrens

Gill Bejerano

Hugo J. Bellen

Jimmy Bennett

Beverly Berg-Rood

Jonathan A. Bernstein

Gerard T. Berry

Anna Bican

Stephanie Bivona

Elizabeth Blue

John Bohnsack

Devon Bonner

Lorenzo Botto

Brenna Boyd

Lauren C. Briere

Gabrielle Brown

Elizabeth A. Burke

Lindsay C. Burrage

Manish J. Butte

Peter Byers

William E. Byrd

John Carey

Olveen Carrasquillo

Thomas Cassini

Ta Chen Peter Chang

Sirisak Chanprasert

Hsiao-Tuan Chao

Ivan Chinn

Gary D. Clark

Terra R. Coakley

Laurel A. Cobban

Joy D. Cogan

Matthew Coggins

F. Sessions Cole

Heather A. Colley

Heidi Cope

Rosario Corona

William J. Craigen

Andrew B. Crouse

Michael Cunningham

Precilla D’Souza

Hongzheng Dai

Surendra Dasari

Joie Davis

Jyoti G. Dayal

Esteban C. Dell'Angelica

Patricia Dickson

Katrina Dipple

Daniel Doherty

Naghmeh Dorrani

Argenia L. Doss

Emilie D. Douine

Dawn Earl

David J. Eckstein

Lisa T. Emrick

Christine M. Eng

Marni Falk

Elizabeth L. Fieg

Paul G. Fisher

Brent L. Fogel

Irman Forghani

William A. Gahl

Ian Glass

Bernadette Gochuico

Page C. Goddard

Rena A. Godfrey

Katie Golden-Grant

Alana Grajewski

Don Hadley

Sihoun Hahn

Meghan C. Halley

Rizwan Hamid

Kelly Hassey

Nichole Hayes

Frances High

Anne Hing

Fuki M. Hisama

Ingrid A. Holm

Jason Hom

Martha Horike-Pyne

Alden Huang

Sarah Hutchison

Wendy Introne

Rosario Isasi

Kosuke Izumi

Fariha Jamal

Gail P. Jarvik

Jeffrey Jarvik

Suman Jayadev

Orpa Jean-Marie

Vaidehi Jobanputra

Lefkothea Karaviti

Shamika Ketkar

Dana Kiley

Gonench Kilich

Shilpa N. Kobren

Isaac S. Kohane

Jennefer N. Kohler

Susan Korrick

Mary Kozuira

Deborah Krakow

Donna M. Krasnewich

Elijah Kravets

Seema R. Lalani

Byron Lam

Christina Lam

Brendan C. Lanpher

Ian R. Lanza

Kimberly LeBlanc

Brendan H. Lee

Roy Levitt

Richard A. Lewis

Pengfei Liu

Xue Zhong Liu

Nicola Longo

Sandra K. Loo

Joseph Loscalzo

Richard L. Maas

Ellen F. Macnamara

Calum A. MacRae

Valerie V. Maduro

AudreyStephannie Maghiro

Rachel Mahoney

May Christine V. Malicdan

Laura A. Mamounas

Teri A. Manolio

Rong Mao

Kenneth Maravilla

Ronit Marom

Gabor Marth

Beth A. Martin

Martin G. Martin

Julian A. Martínez-Agosto

Shruti Marwaha

Jacob McCauley

Allyn McConkie-Rosell

Alexa T. McCray

Elisabeth McGee

Heather Mefford

J. Lawrence Merritt

Matthew Might

Ghayda Mirzaa

Eva Morava

Paolo Moretti

John Mulvihill

Mariko Nakano-Okuno

Stanley F. Nelson

John H. Newman

Sarah K. Nicholas

Deborah Nickerson

Shirley Nieves-Rodriguez

Donna Novacic

Devin Oglesbee

James P. Orengo

Laura Pace

Stephen Pak

J. Carl Pallais

Christina G.S. Palmer

Jeanette C. Papp

Neil H. Parker

John A. Phillips III

Jennifer E. Posey

Lorraine Potocki

Barbara N. Pusey Swerdzewski

Aaron Quinlan

Deepak A. Rao

Anna Raper

Wendy Raskind

Genecee Renteria

Chloe M. Reuter

Lynette Rives

Amy K. Robertson

Lance H. Rodan

Jill A. Rosenfeld

Natalie Rosenwasser

Francis Rossignol

Maura Ruzhnikov

Ralph Sacco

Jacinda B. Sampson

Mario Saporta

Judy Schaechter

Timothy Schedl

Kelly Schoch

Daryl A. Scott

C. Ron Scott

Elaine Seto

Vandana Shashi

Jimann Shin

Edwin K. Silverman

Janet S. Sinsheimer

Kathy Sisco

Edward C. Smith

Kevin S. Smith

Lilianna Solnica-Krezel

Ben Solomon

Rebecca C. Spillmann

Joan M. Stoler

Kathleen Sullivan

Jennifer A. Sullivan

Angela Sun

Shirley Sutton

David A. Sweetser

Virginia Sybert

Holly K. Tabor

Queenie K.-G. Tan

Amelia L. M. Tan

Arjun Tarakad

Mustafa Tekin

Fred Telischi

Willa Thorson

Cynthia J. Tifft

Camilo Toro

Alyssa A. Tran

Rachel A. Ungar

Tiina K. Urv

Adeline Vanderver

Matt Velinder

Dave Viskochil

Tiphanie P. Vogel

Colleen E. Wahl

Melissa Walker

Stephanie Wallace

Nicole M. Walley

Jennifer Wambach

Jijun Wan

Lee-kai Wang

Michael F. Wangler

Patricia A. Ward

Daniel Wegner

Monika Weisz Hubshman

Mark Wener

Tara Wenger

Monte Westerfield

Matthew T. Wheeler

Jordan Whitlock

Lynne A. Wolfe

Kim Worley

Changrui Xiao

Shinya Yamamoto

John Yang

Zhe Zhang

Stephan Zuchner
